# Supplementary material for: Cost-effectiveness of advanced life support and prehospital critical care for out-of-hospital cardiac arrest in England: a decision analysis model
Source: BMJ Open. 2019 Jul 24;9(7):e028574. doi: 10.1136/bmjopen-2018-028574 (PMC6661553; doi:10.1136/bmjopen-2018-028574)
Supplement: Supplementary data [file bmjopen-2018-028574supp001.pdf]

Appendix 1. Search strategy and selection of publications for the synthesis of in-hospital costs of treatment for out-of-hospital cardiac arrest.

We undertook a focused systematic review of the literature regarding the costs of in-hospital treatment for out-of-hospital cardiac arrest (OHCA). Inclusion criteria were any descriptions of costs of in-hospital treatments in adult with non-traumatic OHCA, in the United Kingdom (UK). We did not exclude any particular method. If no data from the UK was available, data from Australia or other European countries was deemed the next best option.

PubMed was searched using the search string *(((cost) OR economics) OR expenditure) AND "cardiac arrest"*, with search results limited to the last 10 years and in English.

One reviewer undertook the screening, and selection of publications according to the above inclusion criteria. See Figure 1 for a flow chart of the search results.

Figure 1. Flow chart of the selection of publications. Search results from PubMed on 22 November 2017.

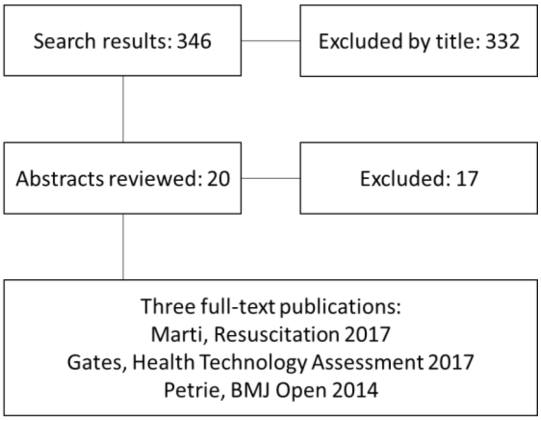

Twelve of the publications for which abstracts were reviewed were excluded as they were undertaken outside the UK, and were therefore expected to have significantly different cost and clinical parameters. The other five excluded studies did not include relevant costs. Of the three full-text publications reviewed, the studies by Marti et al. and Gates et al. related to the same randomised controlled trial of a mechanical chest compression device. Hospital treatment cost estimates were based on the length of stay of patients on the Intensive Care Unit (ICU) and on regular hospital wards as well as Emergency Department (ED) and outpatient costs. In contrast, Petrie et al. included

additional significant costs of cardiac interventions, such as percutaneous coronary intervention (PCI), pacemaker insertion or coronary artery bypass graft surgery (CABG), resulting in an overall higher and probably more accurate estimate of hospital costs for OHCA. We therefore used the data from Petrie et al., including the study's open access source data, in combination with the Department of Health NHS Reference Costs 2015-16, for estimating the costs of in-hospital treatment. Post-discharge costs were extracted from Gates et al.

Due to the limited available evidence and the focused nature of this review, we did not undertake a formal assessment of the risk of bias, synthesis of evidence or a detailed description of the included studies.
