# Supplementary material for: Cost-effectiveness of advanced life support and prehospital critical care for out-of-hospital cardiac arrest in England: a decision analysis model
Source: BMJ Open. 2019 Jul 24;9(7):e028574. doi: 10.1136/bmjopen-2018-028574 (PMC6661553; doi:10.1136/bmjopen-2018-028574)
Supplement: Supplementary data [file bmjopen-2018-028574supp002.pdf]

Appendix 2. Search strategy and selection of publications for the synthesis of quality and length of life after discharge from hospital following out-of-hospital cardiac arrest.

We undertook a focused systematic review of the literature regarding the length and quality of life after survival from out-of-hospital cardiac arrest (OHCA). Inclusion criteria were adult non-traumatic OHCA and outcomes of either length or quality of life measured in utility. Where available, publications using data from the United Kingdom (UK) were included; where no UK data were available, data from North America or Australia were deemed sufficient. We did not exclude any particular methods.

We searched PubMed, using the search string *(((quality of life[MeSH Terms]) OR utility) OR life expectancy[MeSH Terms]) AND cardiac arrest[MeSH Terms]*, with search results limited to the last 10 years.

One reviewer undertook the screening, and selection of publications according to the above inclusion criteria. See Figure 1 for a flow chart of the search results.

Figure 1. Focused literature search for data on length and quality of life following OHCA.

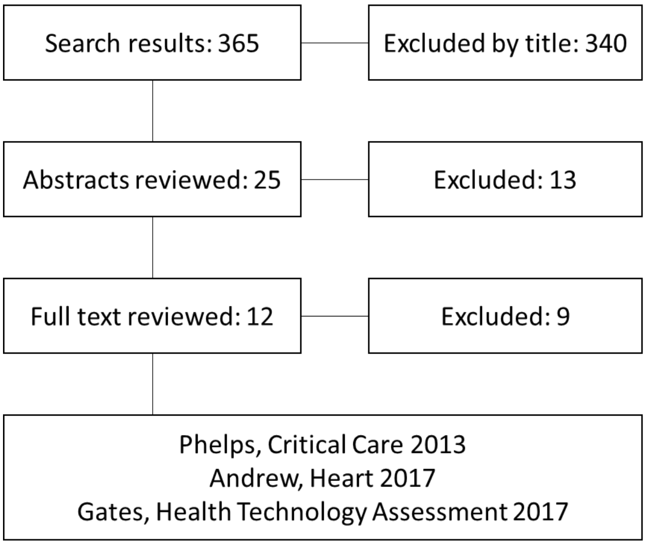

The two questions required for the decision tree and Markov model were

- What is the percentage of Cerebral Performance Category (CPC) 1-2 and CPC 3-4 amongst OHCA patients discharged from hospital?

- What is the quality and length of life of survivors of OHCA with CPC 1-2 and CPC 3-4, respectively?

Percentage of CPC 1-2 and CPC 3-4 at discharge from hospital

Gates et al. provided the most current and relevant information in their randomised controlled trial and embedded economic analysis of mechanical chest compressions during OHCA in the UK. CPC was measured at three months after discharge from hospital, with 245 of 272 (90%) of patients achieving a CPC 1-2. This is somewhat higher than the 78% and 85% of CPC 1-2 survivors reported by Petrie et al. and Phelps et al. in a UK and Australian patient population, respectively. However, both these studies measured CPC at hospital discharge, rather than at three months after discharge. Patients discharged from hospital after OHCA with CPC 3-4 have a significantly higher mortality in the first months post discharge when compared to those discharged with CPC 1-2. Additional data from Phelps et al. at twelve months post discharge demonstrate that, of the 804 patients surviving to one year post discharge, 90% were discharged from hospital with CPC 1-2. We therefore concluded that 85% was a robust estimate for the percentage of OHCA survivors discharged with CPC 1-2.

Length of life after discharge from hospital

Phelps et al. also provide the most detailed description of long-term survival following OHCA, according to CPC at discharge from hospital, including person-years for the first year and the following four years after hospital discharge. We used these data to model mortality during the first 5 years after discharge from hospital following OHCA. Andrew et al. and Phelps et al. showed that survival rates after 5 years following discharge from hospital after OHCA were the same as those of the standard population. Mortality rates were therefore obtained from the National Life Tables for England and applied to the patient cohort from five years onwards. This resulted in an overall survival rate of 65.0% and 50.0% (CPC1-2) and 36.0% and 27.8% (CPC3-4) at 10 and 15 years post discharge, respectively.

Quality of life after discharge from hospital

In their RCT which recruited a total of 4,471 patients with OHCA between 2010 and 2013 in four UK ambulance services, Gates et al. reported health-related quality of life (HRQL) using the EuroQol – 5 dimension descriptive system (EQ-5D). The EQ-5D has been validated for measuring HRQL and for its use in economic evaluations in a variety of conditions, including cardiac arrest. Gates et al. converted their findings to health-state utilities using the UK tariff. Using a combination of decision tree and Markov model similar to that employed in this research, they calculated a utility 0.75 and 0.47 for the CPC 1-2 and CPC 3-4 groups, respectively. The use of EQ-5D for the calculation of utility is recommended by the National Institute for Health and Care Excellence.

Due to the limited available evidence and the focused nature of this review, we did not undertake a formal assessment of the risk of bias, synthesis of evidence or a detailed description of the included studies.
