# Supplementary material for: Cost-effectiveness of advanced life support and prehospital critical care for out-of-hospital cardiac arrest in England: a decision analysis model
Source: BMJ Open. 2019 Jul 24;9(7):e028574. doi: 10.1136/bmjopen-2018-028574 (PMC6661553; doi:10.1136/bmjopen-2018-028574)
Supplement: Author's manuscript [file bmjopen-2018-028574.draft_revisions.pdf]

BMJ Open is committed to open peer review. As part of this commitment we make the peer review history of every article we publish publicly available.

When an article is published we post the peer reviewers' comments and the authors' responses online. We also post the versions of the paper that were used during peer review. These are the versions that the peer review comments apply to.

The versions of the paper that follow are the versions that were submitted during the peer review process. They are not the versions of record or the final published versions. They should not be cited or distributed as the published version of this manuscript.

BMJ Open is an open access journal and the full, final, typeset and author-corrected version of record of the manuscript is available on our site with no access controls, subscription charges or pay-per-view fees (http://bmjopen.bmj.com).

If you have any questions on BMJ Open's open peer review process please email info.bmjopen@bmj.com

BMJ Open

Cost-effectiveness of Advanced Life Support or prehospital critical care for out-of-hospital cardiac arrest: A decision analysis model

Journal:	*BMJ Open*
Manuscript ID	bmjopen-2018-028574
Article Type:	Research
Date Submitted by the Author:	17-Dec-2018
Complete List of Authors:	von Vopelius-Feldt, Johannes; The University of the West of England, Faculty of Health and Applied Sciences; University Hospitals Bristol NHS Foundation Trust, Academic Department of Emergency Care<br>Powell, Jane; University of the West of England, Bristol, Centre for Public Health and Wellbeing<br>Benger, Jonathan; The University of the West of England, Faculty of Health and Applied Sciences; University Hospitals Bristol NHS Foundation Trust, Academic Department of Emergency Care
Keywords:	ACCIDENT & EMERGENCY MEDICINE, Adult intensive & critical care < INTENSIVE & CRITICAL CARE, CARDIOLOGY

SCHOLARONE™
Manuscripts

Cost-effectiveness of Advanced Life Support or prehospital critical care for out-of-hospital cardiac arrest: A decision analysis model

AUTHORS

Johannes von Vopelius-Feldt[1,2] (ORCiD ID 0000-0002-6389-289X)

Jane Powell[3] (ORCiD ID 0000-0002-1828-1184)

Jonathan Benger[1,2] (ORCiD ID 0000-0001-6131-0916)

ASSOCIATIONS

1) Faculty of Health and Applied Sciences, University of the West of England, Bristol, United Kingdom

2) Academic Department of Emergency Care, University Hospitals Bristol NHS Foundation Trust, Bristol, United Kingdom

3) Centre for Public Health and Wellbeing, University of the West of England, Bristol, United Kingdom

CORRESPONDING AUTHOR

Johannes von Vopelius-Feldt

Emergency Care Research Group

1H14, Glenside Campus

Blackberry Hill, BS16 1DD Bristol

University of the West of England, Bristol

johannes.vonvopelius-feldt@uwe.ac.uk

Phone: 0044 (0)7958064069

WORD COUNT

3,141

For peer review only - http://bmjopen.bmj.com/site/about/guidelines.xhtml

ABSTRACT

Objectives

This research aimed to answer the following questions: What are the costs of prehospital Advanced Life Support (ALS) or prehospital critical care for out-of-hospital cardiac arrest (OHCA)? What is the cost-effectiveness of prehospital ALS? What improvement in survival rates from OHCA would prehospital critical care need to achieve in order to be cost-effective?

Setting

A single NHS ambulance service and a charity funded prehospital critical care service.

Participants

The patient population for which this economic analysis applies is adult, non-traumatic OHCA.

Methods

We combined data from previously published research with data provided by a regional ambulance service and air ambulance charity to create a decision analysis model of ALS and prehospital critical care for OHCA. To reflect the uncertainty in the underlying data, we used probabilistic and two-way sensitivity analyses.

Results

Costs of prehospital ALS and prehospital critical care were £347 and £1,711 per patient, respectively. When costs and outcomes of prehospital, in-hospital and post-discharge phase of OHCA care were combined, prehospital ALS was estimated to be cost-effective at £11,407 per quality-adjusted life year (QALY). In order to be cost-effective in addition to ALS, prehospital critical care for OHCA would need to achieve a minimally economically important difference (MEID) in survival to hospital discharge of 3-5%.

Conclusions

This is the first economic analysis to address the question of cost-effectiveness of prehospital critical care following OHCA. While costs of either prehospital ALS and/or critical care per patient with OHCA are relatively low, significant costs incur during hospital treatment and after discharge in patients who survive. Knowledge of the MEID for prehospital critical care can guide future research in this field.

Trial registration

ISRCTN registry ID ISRCTN18375201

For peer review only - http://bmjopen.bmj.com/site/about/guidelines.xhtml

STRENGTHS AND LIMITATIONS OF THIS STUDY

- This is the first study to describe the cost-effectiveness of the complete care pathway for out-of-hospital cardiac arrest in England
- This study uses a combination of primary and secondary data
- The decision analysis model includes a combination of probabilistic and two-way sensitivity analyses to address the complex question of cost-effectiveness of prehospital critical care in a transparent manner
- A limitation of this study is that is based on theoretic modelling only, with uncertainty in the underlying data being reflected in the methods

FUNDING

This work is funded by a National Institute for Health Research (NIHR) doctoral research fellowship for Johannes von Vopelius-Feldt (DRF-2015-08-040). The funder is not involved in the design of the study or collection, analysis and interpretation of data, or in writing the manuscript. The views expressed are those of the author(s) and not necessarily those of the NHS, the NIHR or the Department of Health.

COMPETING INTEREST

JVVF and JB work as prehospital doctors with a regional prehospital critical care team.

INTRODUCTION

Rates of survival following out-of-hospital cardiac arrest (OHCA) remain low in the UK, with an overall survival rate to hospital discharge of 7.9%.[1] One area of potential improvement is the provision of prehospital care by the Emergency Medical Services (EMS).[2] The current standard of prehospital care in the UK is the provision of Advanced Life Support (ALS) by appropriately trained paramedics.[3] In an attempt to improve survival rates following OHCA, prehospital critical care teams (CCTs) are dispatched to OHCA in some regions in the UK, in addition to the standard ALS response.[4, 5] CCTs consist of specialist paramedics and/or doctors, with extended skills and competences, which we previously described in detail.[6] They are frequently based on air ambulances, with funding received variably through charitable donations and/or the National Health Service (NHS).[7]

The cost-effectiveness of ALS care provided by paramedics or physicians has been studied previously.[8, 9] However, generalisability of this evidence to current UK practice is limited due to the time elapse since the research was undertaken and considerable differences in EMS configurations. The cost-effectiveness of prehospital critical care for OHCA has not been researched in sufficient detail

For peer review only - http://bmjopen.bmj.com/site/about/guidelines.xhtml

For peer review only

to guide funding decisions. A recently published systematic review of the effectiveness of CCTs for OHCA showed a potential small to moderate survival benefit but also identified issues limiting internal and external validity of the included observational studies.[10]

Economic analyses have been undertaken in regards to the cost-effectiveness of helicopter-based EMS care (HEMS) for trauma, with mixed results.[11, 12] To our knowledge, no economic analysis exists which addresses the question of cost-effectiveness of prehospital critical care for OHCA. In addition, a recent study of stakeholders' views of prehospital care for OHCA revealed contrasting opinion of whether current NHS funding of prehospital ALS care for OHCA represents an appropriate distribution of limited healthcare resources in the UK.[13] Adding to the complexity of cost-effectiveness of prehospital ALS or critical care for OHCA is the fact that the majority of costs occur along the patient care pathway in hospital or post-discharge.[14, 15]

In the absence of robust data regarding the effectiveness of prehospital ALS or critical care for OHCA in the UK setting, this study addresses the following questions

- What are the costs of prehospital ALS and prehospital care for OHCA in the UK?
- What is the cost-effectiveness of the current OHCA treatment pathway from prehospital ALS care to post-discharge costs?
- What improvement in survival rates from OHCA would prehospital critical care need to achieve in order to be cost-effective?

METHODS

We combined data from previously published research with costs provided by a regional EMS provider and air ambulance charity to create a decision analysis model. After an initial review of the literature, the final model contained a combination of decision tree model for prehospital and hospital costs and outcomes and a Markov model for post-discharge costs and outcomes (see appendix 1).

Perspective and time horizon

In keeping with the recommendations of the National Institute for Health and Care Excellence (NICE), the perspective on costs chosen for this economic evaluation was that of the National Health Service (NHS) and personal social services.[16] The perspective on outcomes included all direct health effects on patients, namely length and quality of life (quality-adjusted life years, QALYs) with a corresponding long-term time horizon.

For peer review only - http://bmjopen.bmj.com/site/about/guidelines.xhtml

Clinical setting

The economic analysis was undertaken in the section of an NHS ambulance trust in England which was covered by a critical care team. It covers an area of 2.930 square miles with a population of approximately 2.5 million people. The area is largely suburban or rural with one metropolitan area and two urban areas. The overall population density is 860/square mile. The patient population is adult, non-traumatic OHCA.

Alternative interventions – Advanced life support and prehospital critical care

The NHS ambulance trust provided ALS paramedic care, using a combination of first responders, rapid response vehicles and ambulance. The section of the ambulance trust included in this analysis contained 27 ambulance stations with a total of approximately 800 ALS paramedics (employed part- or full-time) as well as emergency care assistants (ECAs) and ambulance technicians (data provided by ambulance trust). The median response time for OHCA during the study period was 7.4min (IQR 4.9min – 11.4min) and care on scene was provided by a mean of 2.43 core resources (paramedic-staffed double-crewed ambulances and/or rapid response vehicles). The prehospital critical care service was funded largely through an air ambulance charity but also received support from the ambulance trust. It provided prehospital critical care cover from 7.00am to 1.00am, with access to a helicopter and rapid response vehicles. The team consisted of specialist paramedics in critical care and prehospital critical care doctors. The service responded to OHCAs in addition to the usual ambulance trust's ALS response.

Identification and synthesis of evidence

Costs

Financial data was obtained from the ambulance trust and air ambulance charity for the financial year 2015/16. This included pay for clinical and non-clinical staff as well as costs of dispatch infrastructure, ambulance stations, vehicles, fuel, medical supplies, communications equipment, community first responders and 3rd party costs. The amount of funding spent on OHCA was calculated as the number of OHCA cases divided by the number of cases of any aetiology. Calculating the proportion of resources spent on OHCA in this fashion distributes the costs of waiting time of resources in between emergency proportionally between OHCA cases and those of other aetiology.[17] The costs of in-hospital and post-discharge treatment were synthesised from relevant recent publications, identified by a focused systematic search of the literature (appendix 2).[14, 15, 18]

Outcomes

After a literature review (appendix 3), the following outcomes of interest were included in the decision analysis model (appendix 1): survival to arrival at hospital; survival to ICU admission; survival to

For peer review only - http://bmjopen.bmj.com/site/about/guidelines.xhtml

hospital discharge; cerebral performance category (CPC) at discharge from hospital; 5-year survival following hospital discharge; annual mortality and quality of life. Prehospital outcomes after OHCA with ALS care were based on a one-year data sample provided by the participating ambulance trust (2016), while in-hospital outcomes were based on recent literature of OHCA in the UK.[19-21] For prehospital critical care, a range of effect sizes were simulated in two-way sensitivity analyses.[10] This approach was chosen to transparently reflect the considerable uncertainty of the effectiveness of prehospital critical care for OHCA in current research.[10] Post-discharge outcomes for the first five years after hospital discharge were retrieved from publications identified through a further focused systematic search of the literature (appendix 3).[15, 22] Further long-term outcomes (quality of life) were modelled according to survival rates provided by the UK's Office for National Statistics.[23, 24]

Application of values and distributions to the model

Prior to inclusion in the model, all costs were adjusted to Pound Sterling for the financial year 2016-17 according to the UK Hospital and Community Health Services (HCHS) pay and prices index.[25] In keeping with NICE guidelines, an annual discount rate of 3.5% was applied to costs and effects which occurred after the first year following discharge from hospital.[16] Table 1 provides a comprehensive list of all model parameters, based on the sources of data described in the previous section. The confidence intervals or interquartile ranges of the data were transferred to sampling distributions.[26] In keeping with previous recommendations, we used Gamma distributions for costs and Beta distributions for probabilities.[26]

For peer review only

For peer review only - http://bmjopen.bmj.com/site/about/guidelines.xhtml

Table 1. Values and distributions of parameters of the decision tree and Markov model; all costs adjusted for inflation to the financial year 2016-17

Parameters	Cohort / patient group	Mean value (95% confidence interval)	Distribution	Source
**Prehospital (decision tree model)**				
Costs of prehospital care	ALS	£347	Gamma	Own data
	Critical care (in addition to ALS)	£2,058 (£1,711 critical care plus £347 ALS care)	Sensitivity analysis	
p survival to hospital	ALS	25.8% (24.6% – 27.0%)	Beta	Simulated data
	Critical care	24.5% to 31.0%	Sensitivity analysis	
**In-hospital (decision tree model)**				
Costs of ED treatment		£377 (£355 – £399)	Gamma	Department of Health [27]
p ICU admission*		0.65 (0.58 – 0.71)	Beta	Benger [19]
Costs of ICU treatment (daily) and length of stay on ICU (LOS)	Survival to hospital discharge	£1,745 (£1,654 – £1,836)<br>LOS (days) 5.7 (5.4 – 6.0)	Gamma	Petrie [14]<br>Nolan [20]
	In-hospital death	£1,768 (£1,668 – £1,868)<br>LOS (days) 2.4 (2.3 – 2.5)		
Cost of non-ICU treatment (total)	Survival to hospital discharge	£12,823 (£10,123 – £15,523)	Gamma	Petrie [14]<br>Nolan [20]
	In-hospital death	£3,835 (£3,045 – £4,625)		
p survival to hospital discharge**	ALS	9.0% (8.2% - 9.7%)	Beta	Simulated data
	Critical care	10.0% – 15.0%	Sensitivity analysis	
**Post-discharge (decision tree model)**				
p survival with CPC1-2***		0.85 (0.826 – 0.871)	Beta	Phelps [22]
p 5-year survival	CPC1-2	0.740 (0.709 – 0.768)	Beta	Phelps [22]
	CPC3-4	0.408 (0.332 – 0.489)		
Person-years per death within 5 years	CPC1-2	1.827 (1.750 – 1.878)	Gamma	Phelps [22]
	CPC3-4	0.313 (0.254 – 0.375)		
Utility	CPC1-2	0.75 (0.70 – 0.80)	Beta	Gates [15]
	CPC3-4	0.47 (0.42 – 0.52)		
Annual healthcare costs	CPC1-2	£3,358	Gamma	Gates [15]
	CPC3-4	£43,670		
**Long-term outcomes (Markov model)**				
p annual mortality after 5 years post discharge	CPC1-2<br>CPC3-4	National age-adjusted mortality rates	Exact values	Office for National Statistics [23], Andrew [24]

*of survivors to hospital **of admissions to ICU ***of survivors to hospital discharge
ALS: Advanced Life Support, p: probability, ED: emergency department, ICU: intensive care unit, LOS: length of stay, CPC: Cerebral Performance Category

For peer review only

For peer review only - http://bmjopen.bmj.com/site/about/guidelines.xhtml

Sensitivity analyses

As the analysis was based largely on secondary data, we undertook a probabilistic sensitivity analysis of the decision analysis model, to reflect the underlying uncertainty in the data.[26] We ran 1,000 iterations of the decision analysis model in a Monte Carlo simulation. For each iteration, the values of all model parameters were drawn at random from the sampling distributions described in table 1. In addition, the probabilities of survival to hospital admission and survival to hospital discharge after prehospital critical care were subject to a two-way sensitivity analysis, reflecting a range of potential effects based on a recent systematic review.[10] All analysis was undertaken in TreeAge (Version 2017).

Patient and public involvement

A Patient and Public Involvement (PPI) Group was consulted during the application and planning process of the doctoral research fellowship which underlies this research. In addition, the PPI group's as well as other relevant stakeholders' views expressed during previous research[13] informed the methods of this research. Results will be disseminated to all stakeholders who participated, through an executive summary of this research.

RESULTS

Prehospital costs

Table 2 gives an overview of the costs of prehospital ALS care and prehospital critical care for OHCA. The average cost of a patient with OHCA being attended by ALS paramedics is £347 with an incremental cost of £1,711 for the prehospital critical care team.

For peer review only

Table 2. Prehospital treatment costs for out-of-hospital cardiac arrest

Total expenditure 2015-16 (in £)		
	**ALS care**	**Critical care**
Staff costs	36,732,844	665,293
Vehicles (including fuel)	8,145,245	13,659
Helicopter (including fuel)	-	1,318,432
Buildings	2,705,442	37,693
Equipment	1,681,383	63,296
Other	6,261,901	37,752
Dispatch centre	2,226,955	45,000
**Total**	**57,753,770**	**2,181,125**
**Expenditure per OHCA 2015-16 (in £)**		
**Percentage of resources devoted to OHCA**	0.85%	30%
**Number of OHCA patients**	1412	388
**Cost per OHCA patient**	**347**	**1,711**

Cost-effectiveness of Advanced Life Support for OHCA

The estimated cost-effectiveness of the current standard pathway for OHCA, including prehospital ALS, hospital admission, ICU and non-ICU treatment, as well as post-discharge healthcare costs, is £11,407/QALY (median, IQR £6,840 /QALY – £16,863/QALY). Figure 1 shows the corresponding scatterplot with results from 1,000 iterations of the probabilistic sensitivity analysis.

Incremental cost-effectiveness of prehospital critical care for OHCA

Figure 2 shows the cost-effectiveness acceptability curves for a number of potential treatment effects of prehospital critical care following OHCA. For this model, the ratio of rates of survival to hospital and survival to hospital discharge in the prehospital critical care treatment arm was kept constant. When compared to ALS with a survival to hospital rate of 25.8% and a rate of survival to hospital discharge of 9%, prehospital critical care needs to achieve a 2% or 3% absolute increase in survival to hospital discharge to have a 48% or 72% probability of being cost effective at a willingness-to-pay (WTP) threshold of £20,000 per QUALY, respectively. With a lower absolute difference in survival rates of 1%, prehospital critical care has a less than 1% probability of being cost effective at the same WTP threshold. Higher absolute differences in survival of 4%, 5% and 6% result in probabilities of cost-effectiveness at the WTP threshold of £20,000 of 84%, 90% and 93%, respectively.

The results of the analysis presented in figure 2 assume that the rates of survival to hospital increase proportionally with the rates of survival to hospital discharge. However, as the majority of costs for

For peer review only - http://bmjopen.bmj.com/site/about/guidelines.xhtml

the care of OHCA is generated by in-hospital treatments, variations in the rate of survival to hospital can have considerable influence on costs, independently of later survival to hospital discharge. Table 3 provides estimates and interquartile ranges for possible combinations of survival to hospital and survival to hospital discharge rates. Table 4 presents a further sensitivity analysis of the effects of varying costs of prehospital critical care and rates of survival to hospital discharge.

Table 3. Incremental cost-effectiveness of prehospital critical care for OHCA (in £ per quality-adjusted life year), for different rates of survival to hospital and survival to hospital discharge, compared to ALS.

**Difference in rates of survival to hospital**	**Difference in rates of survival to hospital discharge (median, 95% CI)**					
	**+1%**	**+2%**	**+3%**	**+4%**	**+5%**	**+6%**
**-5%**	25,000 (16,500 – 46,000)	18,000 (12,500 – 28,500)	15,500 (11,000 – 23,500)	14,500 (10,500 – 21,000)	Not achievable	Not achievable
**0%**	**27,500 (20,000 – 47,500)**	19,000 (14,500 – 30,000)	16,500 (12,500 – 24,500)	15,000 (11,500 – 22,000)	14,000 (11,000 – 20,000)	13,500 (10,500 – 19,000)
**+5%**	30,500 (21,500 – 53,000)	**20,500 (15,500 – 33,500)**	17,000 (13,000 – 26,500)	15,500 (12,000 – 23,000)	14,500 (11,500 – 21,500)	14,000 (11,000 – 20,000)
**+10%**	33,500 (22,500 – 61,000)	22,000 (16,000 – 37,500)	**18,000 (14,000 – 28,500)**	**16,500 (12,500 – 24,500)**	15,000 (12,000 – 22,000)	14,500 (11,500 – 21,000)
**+15%**	36,500 (23,000 – 70,500)	23,500 (17,000 – 42,000)	20,000 (14,500 – 32,000)	17,000 (13,000 – 26,500)	**16,000 (12,500 – 23,500)**	**15,000 (11,500 – 22,000)**
**+20%**	39,500 (23,500 – 79,500)	24,500 (17,000 – 46,000)	20,000 (15,000 – 34,000)	17,500 (13,500 – 29,000)	16,500 (12,500 – 25,500)	15,500 (12,000 – 23,000)

Light and darker shades of grey indicate most likely combination of differences in rates of survival to hospital and survival to hospital discharge. All costs are in £ and have been rounded to the nearest £500 value.

For peer review only - http://bmjopen.bmj.com/site/about/guidelines.xhtml

Table 4. Incremental cost-effectiveness of prehospital critical care for OHCA (in £ per quality-adjusted life year), for a range of costs of prehospital critical care and different rates of survival to hospital discharge, compared to ALS.

Difference in costs of prehospital care	Difference in rates of survival to hospital discharge (median, 95% CI)					
	**+1%**	**+2%**	**+3%**	**+4%**	**+5%**	**+6%**
**-50%**	20,000 (16,000 – 33,500)	16,000 (13,000 – 25,500)	15,000 (12,000 – 23,000)	14,000 (11,500 – 21,500)	14,000 (11,000 – 21,000)	13,500 (11,000 – 20,500)
**-20%**	25,000 (19,000 – 43,000)	18,500 (14,500 – 30,500)	16,500 (13,000 – 26,000)	15,500 (12,500 – 24,000)	15,000 (12,000 – 23,000)	14,500 (11,500 – 22,000)
**0%**	**28,000 (21,500 – 50,500)**	**20,000 (16,000 – 33,500)**	**17,500 (14,000 – 28,500)**	**16,000 (13,000 – 25,500)**	**15,500 (12,500 – 24,000)**	**15,000 (12,000 – 23,000)**
**+20%**	31,500 (24,000 – 57,500)	22,000 (17,000 – 37,000)	18,500 (14,500 – 30,500)	17,000 (13,500 – 27,000)	16,000 (13,000 – 25,500)	15,500 (12,500 – 24,000)
**+50%**	36,000 (27,500 – 67,500)	24,00 (18,500 – 41,500)	20,000 (16,000 – 33,500)	18,000 (14,500 – 30,000)	17,000 (13,500 – 27,000)	16,000 (13,000 – 25,500)

All costs are in £ and have been rounded to the nearest £500 value.

eer review only

DISCUSSION

In this decision model analysis, the cost-effectiveness of prehospital ALS care or prehospital critical care for OHCA was determined to a considerable degree by their effects on short and long-term survival, with the majority of costs incurring during hospital treatment or post-discharge.

Prehospital costs vs later costs

Given the complexity of the economic analysis of the whole OHCA pathway, it is worth first looking at prehospital resource use only. The ambulance service described in this analysis had an annual spending of over 50 million pounds sterling. However, during the study period, patients with OHCA only represented 0.22% of all emergency calls attended. Even with the requirement for more resources per OHCA patient compared to most other prehospital conditions, only 0.82% of the annual spending was allocated to OHCAs, at an estimated cost of £347 per OHCA. This might seem a rather low amount of money to be spent on the immediate care for a life or death situation.[13]

For peer review only - http://bmjopen.bmj.com/site/about/guidelines.xhtml

In contrast, the annual costs of providing a helicopter-based prehospital critical care team, covering the same geographic area was relatively low, at just over 2 million pounds per year. Due to the fact that the critical care team attended much fewer patients (approximately 1,200 during the study period) with a higher proportion of OHCAs (30%), the incremental costs of prehospital critical care per OHCA were estimated at £1,711. Given the gravity of the acute situation of an OHCA, this incremental cost might be considered an effective use of EMS resources.

Focusing only on prehospital costs of OHCA would make comparison to other healthcare interventions difficult, and would ignore the importance of costs accumulating further along the OHCA pathway. For patients with OHCA, where resuscitation is unsuccessful and who are declared dead on scene, no further costs occur. However, a large proportion (25.8% in this cohort) of patients with OHCA survive the prehospital phase of their care and are admitted to a hospital. We estimated the costs of in-hospital treatment to be approximately £22,000 for patients surviving to hospital discharge and £8,500 for patients who die in hospital.[14, 20] The major contributors to these costs are ICU-bed days (all patients) and interventions such as PPCI, ICD implantation or coronary artery bypass graft surgery (survivors only).[14] Further costs can accumulate after hospital discharge, most importantly the long-term care services required for the small proportion of patients who survive to hospital discharge with poor neurological function (over £40,000 per year).[15]

Cost-effectiveness of Advanced Life Support for OHCA

We estimated the cost-effectiveness of paramedic-delivered ALS for OHCA to be approximately £11,500 per QALY. With the upper limit of the interquartile range at approximately £16,800, this makes ALS for OHCA almost certainly cost-effective at a NICE WTP threshold of £20,000 per QUALY.[16]

While a number of previous publications address the cost-effectiveness of individual aspects of prehospital care or hospital care for OHCA,[28-30] only few address the cost-effectiveness of ALS for OHCA in the context of the complete patient pathway. Naess et al.[31] estimated the cost-effectiveness of ALS for OHCA in Norway to be approximately £6,000 per QUALY in 2004. This much lower estimate is likely due to inflation since the year of publication but also development of in-hospital cardiac arrest care with higher rates of interventions in modern OHCA care, and the costs associated with this.[20, 32] More recently, Ginsberg et al.[33] reported the cost-effectiveness of ALS for OHCA to be $28,864 per Disability Adjusted Life Year (DALY) averted, in an Israeli EMS, corresponding to approximately £15,000 - £28,500 per QALY.[34] Despite some uncertainty of the data underlying this analysis, and taking previous research findings into account, we can be fairly certain that paramedic-delivered ALS is a cost-effective treatment for OHCA in the UK. In addition, In

For peer review only - http://bmjopen.bmj.com/site/about/guidelines.xhtml

terms of £ per QUALY, it compares favourably with a range of interventions currently funded by the NHS.[35, 36]

Costs of prehospital critical care

As the current observational research describing the effectiveness of prehospital critical care for OHCA is inconsistent and likely suffers from confounding,[10] we decided not to include it in this probabilistic sensitivity analysis. Instead, we used a range of potential absolute effect sizes on rates of survival to hospital discharge, ranging from 1% to 6%, reflecting the most likely range of effect sizes.[10] This sensitivity analysis also allowed us to determine a minimally economically important difference (MEID) in survival rates which would need to be achieved by prehospital critical care services in order to be cost-effective.

At a WTP threshold of £20,000 per QALY, the MEID is in the range of 3-5% absolute improvement in survival to hospital discharge after OHCA. Any improvement in survival rates after OHCA of less than 3%, caused by prehospital critical care services, is very unlikely to be considered cost-effective within an NHS setting.[16] The exact value for the MEID depends on how certain stakeholders want to be about the cost-effectiveness estimate at a given WTP (figure 2), the rates of survival to hospital admission (table 3) and the exact costs of a given prehospital critical care service (table 4). The MEID can be used to guide sample size calculations of future research and to frame the expectations or aims of stakeholders and prehospital critical care services providing care for OHCA.[13] Interestingly, the MEID of prehospital critical care for OHCA calculated in this analysis equals the minimally clinically important difference (MCID) in survival following OHCA described in previous research.[37]

LIMITATIONS

This economic analysis is based on a theoretical construct of the costs and effects of the care pathway for OHCA. As such, assumptions about events occurring in reality had to be made, and have to be assumed to reflect reality accurately, in order for the model to be internally valid.[26] On the other hand, using a modelling approach allowed us to incorporate a considerable number of data sources and to undertake a range of sensitivity analyses, which improves generalisability.

We did not adapt a micro-costing approach which would have, for example, included the exact number of paramedics at scene for an OHCA or the amount of drugs or equipment used. Such an approach would have likely resulted in an overall more accurate estimate of the costs of ALS care for OHCA, but would have required tremendous efforts to collect and analyse the data. As costs occurring in the later stages of care for OHCA were much higher than during the prehospital phase, a micro-

For peer review only - http://bmjopen.bmj.com/site/about/guidelines.xhtml

costing approach would have been unlikely to change the cost estimate for prehospital care significantly. Reassuringly, the estimated cost of £347 for ALS care for OHCA compares realistically to the NHS National Schedule of Reference Costs of an average of £236 for *'see and treat and convey'* ambulance activities (considering the higher resource use for OHCA patients).[27]

The main driver of costs for prehospital critical care was the helicopter, followed by staffing costs. We are aware of a number of prehospital critical care services which attend OHCAs using only ground vehicles. This would certainly reduce overall costs of the service (and is therefore reflected in the sensitivity analysis) but also the number of OHCAs which the CCT could reach, particular in rural/suburban areas. In regards to the validity of the overall model, there are few previous publications to compare to. Reassuringly, Delgado et al.[38] used a similar decision model analysis approach to examine the potential cost-effectiveness of HEMS for trauma cases in the US. They found that a 3.7% absolute decrease in mortality was required for HEMS to be cost-effective at a WTP of $50,000 (£38,000) per QALY.

This analysis focuses exclusively on adult, non-traumatic OHCA, as OHCA due to trauma or occurring in the paediatric population is much rarer and due to distinctively different pathology. Results should not be extrapolated to these situations.

CONCLUSION

This is the first economic analysis, based on a decision analysis model, to address the question of cost-effectiveness of prehospital critical care following OHCA. While costs of either prehospital ALS and/or critical care per patient with OHCA are relatively low, significant costs incur during hospital treatment and after discharge in patients who survive. Taking the whole OHCA care pathway into account, prehospital ALS as currently delivered in most NHS ambulance trusts is very likely to be cost-effective at a WTP of £20,000 per QALY. For prehospital critical care to be cost-effective at the same WTP threshold, it would need to improve survival from OHCA by approximately 3-5%.

For peer review only - http://bmjopen.bmj.com/site/about/guidelines.xhtml

REFERENCES

1. Hawkes C, Booth S, Ji C, et al. Epidemiology and outcomes from out-of-hospital cardiac arrests in England. *Resuscitation* 2017;110:133-140.
2. Boyce LW, Vliet Vlieland TP, Bosch J, et al. High survival rate of 43% in out-of-hospital cardiac arrest patients in an optimised chain of survival. *Neth Heart J* 2015;23(1):20-5.
3. Brown S, Kumar D, Millins M, et al. UK ambulance services clinical practice guidelines 2016. Bridgewater: Class Professional 2016.
4. von Vopelius-Feldt J, Benger J. Critical care paramedics in England: a national survey of ambulance services. *Eur J Emerg Med* 2014;21(4):301-4.
5. Hyde P, Mackenzie R, Ng G, et al. Availability and utilisation of physician-based pre-hospital critical care support to the NHS ambulance service in England, Wales and Northern Ireland. *Emerg Med J*, 2012;29(3):177-181.
6. von Vopelius-Feldt, J. and J. Benger, Who does what in prehospital critical care? An analysis of competencies of paramedics, critical care paramedics and prehospital physicians. *Emerg Med J* 2014;31(12):1009-13.
7. Association of Air Ambulances Annual Report 2016. Henley in Arden: Association of Air Ambulances 2017.
8. Nichol G, Laupacis A, Stiell IG, et al. Cost-effectiveness analysis of potential improvements to emergency medical services for victims of out-of-hospital cardiac arrest. *Ann Emerg Med* 1996;27(6):711-20.
9. Yen ZS, Chen YT, Ko PC, et al. Cost-effectiveness of different advanced life support providers for victims of out-of-hospital cardiac arrests. *J Formos Med Assoc* 2006;105(12):1001-7.
10. von Vopelius-Feldt J, Brandling J, Benger J. Systematic review of the effectiveness of prehospital critical care following out-of-hospital cardiac arrest. *Resuscitation* 2017;114:40-46.
11. Taylor CB, Stevenson M, Jan S, et al. A systematic review of the costs and benefits of helicopter emergency medical services. *Injury* 2010;41(1):10-20.
12. Snooks HA, Nicholl JP, Brazier JE, et al. The costs and benefits of helicopter emergency ambulance services in England and Wales. *J Public Health Med* 1996;18(1):67-77.

For peer review only - http://bmjopen.bmj.com/site/about/guidelines.xhtml

13. Von Vopelius-Feldt J, Brandling J, Benger J. Variations in stakeholders' priorities and views onrandomisation and funding decisions in out-of-hospital cardiacarrest: An exploratory study. *Health Sci Rep* 2018;1(9):e78.

14. Petrie J, Easton S, Naik V, et al. Hospital costs of out-of-hospital cardiac arrest patients treated in intensive care; a single centre evaluation using the national tariff-based system. *BMJ Open* 2015;5(4):e005797.

15. Gates S, Lall R, Quinn T, et al. Prehospital randomised assessment of a mechanical compression device in out-of-hospital cardiac arrest (PARAMEDIC): a pragmatic, cluster randomised trial and economic evaluation. *Health Technol Assess* 2017;21(11):1-176.

16. National Institute for Health and Care Excellence. Guide to the methods of technology appraisal 2013. www.nice.org.uk/process/pmg9 (accessed 14 December 2018).

17. Lerner EB, Garrison HG, Nichol G, et al. An Economic Toolkit for Identifying the Cost of Emergency Medical Services (EMS) Systems: Detailed Methodology of the EMS Cost Analysis Project (EMSCAP). *Acad Emerg Med* 2012;19(2):210-216.

18. Petrie J, Easton S, Naik V, et al. Data from: Hospital costs of out-of-hospital cardiac arrest patients treated in intensive care; a single centre evaluation using the national tariff-based system. Dryad Digital Repository 2015. www.datadryad.org/resource/doi:10.5061/dryad.k78g1 (accessed 14 December 2018).

19. Benger J, Coates D, Davies S, et al. Randomised comparison of the effectiveness of the laryngeal mask airway supreme, i-gel and current practice in the initial airway management of out of hospital cardiac arrest: a feasibility study. *Br J Anaesth* 2016;116(2):262-8.

20. Nolan JP, Ferrando P, Soar J, et al. Increasing survival after admission to UK critical care units following cardiopulmonary resuscitation. *Crit Care* 2016;20(1):219.

21. Wan X, Wang W, Liu J, et al. Estimating the sample mean and standard deviation from the sample size, median, range and/or interquartile range. *BMC Med Res Methodol* 2014;14:135.

22. Phelps R, Dumas F, Maynard C, et al. Cerebral Performance Category and long-term prognosis following out-of-hospital cardiac arrest. *Crit Care Med* 2013;41(5):1252-7.

23. Office for National Statistics. National Life Tables 2015-2017. UK Statistics Authority 2018. www.ons.gov.uk/peoplepopulationandcommunity/birthsdeathsandmarriages/lifeexpectancies/datasets/nationallifetablesunitedkingdomreferencetables (accessed 14 December 2018).

For peer review only - http://bmjopen.bmj.com/site/about/guidelines.xhtml

24. Andrew E, Nehme Z, Wolfe R, et al. Long-term survival following out-of-hospital cardiac arrest. *Heart* 2017;103(14):1104-1110.

25. Department of Health. HCHS Pay and Price Series 2015/16. http://www.info.doh.gov.uk/doh/finman.nsf/af3d43e36a4c8f8500256722005b77f8/360a47827991d10a80258036002d8d9f?OpenDocument (accessed 14 December 2018).

26. Briggs A, Claxton K, Sculpher M. Decision modelling for health economic evaluation. Oxford: Oxford University Press 2006.

27. Department of Health, Reference Costs 2015-16. https://www.gov.uk/government/collections/nhs-reference-costs (accessed 14 December 2018).

28. Marti J, Hulme C, Ferreira Z, et al. The cost-effectiveness of a mechanical compression device in out-of-hospital cardiac arrest. *Resuscitation* 2017;117:1-7.

29. Moran PS, Teljeur C, Masterson S, et al. Cost-effectiveness of a national public access defibrillation programme. *Resuscitation* 2015;91:48-55.

30. Merchant RM, Becker LB, Abella BS, et al. Cost-effectiveness of therapeutic hypothermia after cardiac arrest. *Circ Cardiovasc Qual Outcomes* 2009;2(5):421-8.

31. Naess AC, Steen PA. Long term survival and costs per life year gained after out-of-hospital cardiac arrest. *Resuscitation* 2004;60(1):57-64.

32. Lai H, Choong CV, Fook-Chong S, et al. Interventional strategies associated with improvements in survival for out-of-hospital cardiac arrests in Singapore over 10 years. *Resuscitation* 2015;89:155-61.

33. Ginsberg GM, Kark JD, Einav S. Cost-utility analysis of treating out of hospital cardiac arrests in Jerusalem. *Resuscitation* 2015;86:54-61.

34. Sassi F. Calculating QALYs, comparing QALY and DALY calculations. *Health Policy Plan* 2006;21(5):402-8.

35. Pharoah PD, Sewell B, Fitzsimmons D, et al. Cost effectiveness of the NHS breast screening programme: life table model. *BMJ* 2013;346:f2618.

36. Hartwell D, Colquitt J, Loveman E, et al. Clinical effectiveness and cost-effectiveness of immediate angioplasty for acute myocardial infarction: systematic review and economic evaluation. *Health Technol Assess* 2005;9(17):1-99.

For peer review only - http://bmjopen.bmj.com/site/about/guidelines.xhtml

37. Nichol G, Brown S, Perkins G, et al. What change in outcomes after cardiac arrest is necessary to change practice? Results of an international survey. *Resuscitation* 2016;107:115-20.

38. Delgado MK, Staudenmayer K, Wang N, et al. Cost-Effectiveness of Helicopter Versus Ground Emergency Medical Services for Trauma Scene Transport in the United States. *Ann Emerg Med* 2013;62(4):351-364.

AUTHORS' CONTRIBUTIONS

JV designed the study protocol, collected and analysed the data and prepared the first draft of the manuscript.

JP reviewed the study protocol, supervised data analysis and reviewed and revised the manuscript.

JB supervised the research project and reviewed and revised the manuscript.

ACKNOWLEDGMENTS

We want to thank the South Western Ambulance Service NHS Foundation Trust and the Great Western Air Ambulance Charity for providing the data for this research. We are particularly grateful to the Patient and Public involvement group and the other participants in our previous research, whose views informed this research.

FIGURE LEGENDS

Figure 1. Scatterplot of the cost-effectiveness of Advanced Life Support for out-of-hospital cardiac arrest

Figure 2. Cost-effectiveness acceptability curves of different treatment effects of prehospital critical care for OHCA, when compared to ALS. Baseline survival to hospital discharge for the ALS cohort is 9.0%.

For peer review only

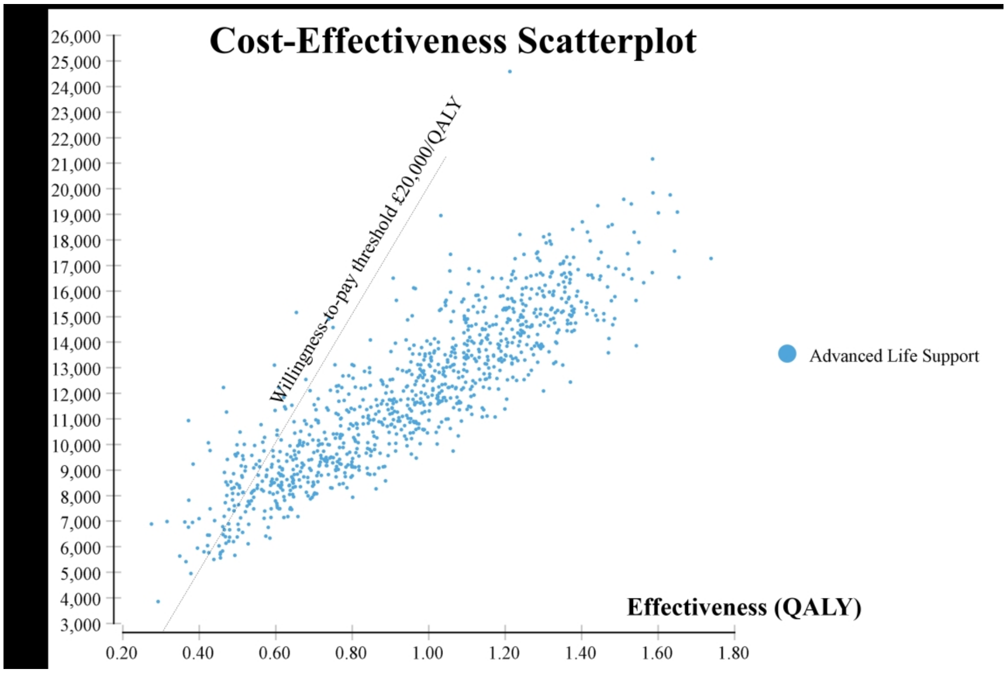

Figure 1. Scatterplot of the cost-effectiveness of Advanced Life Support for out-of-hospital cardiac arrest

250x166mm (300 x 300 DPI)

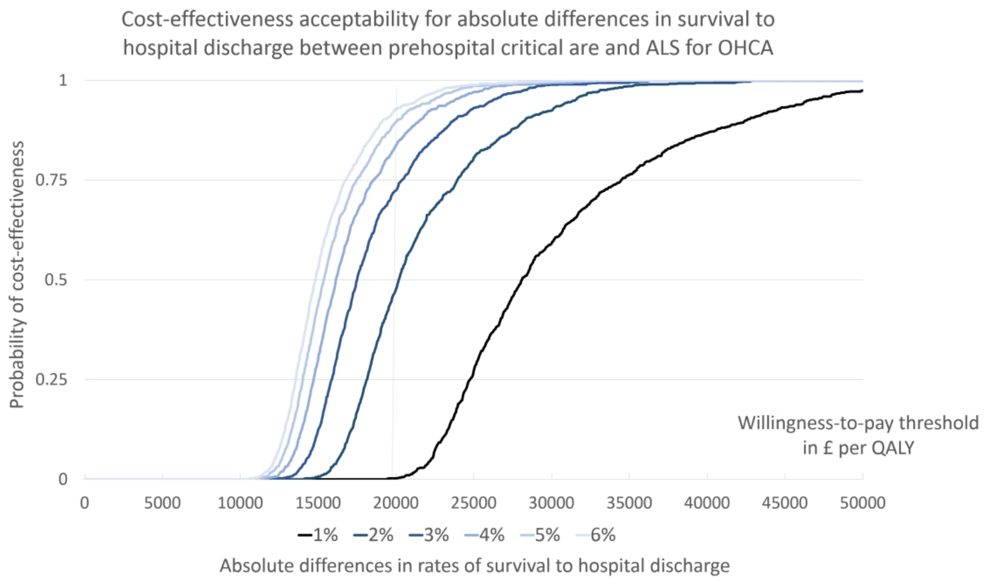

Figure 2. Cost-effectiveness acceptability curves of different treatment effects of prehospital critical care for OHCA, when compared to ALS. Baseline survival to hospital discharge for the ALS cohort is 9.0%.

Appendix 1. Final analytic model of prehospital critical care following out-of-hospital cardiac arrest, combining decision tree and Markov model. Due to limited space, the full pathway is shown for the Advanced Life Support option only.

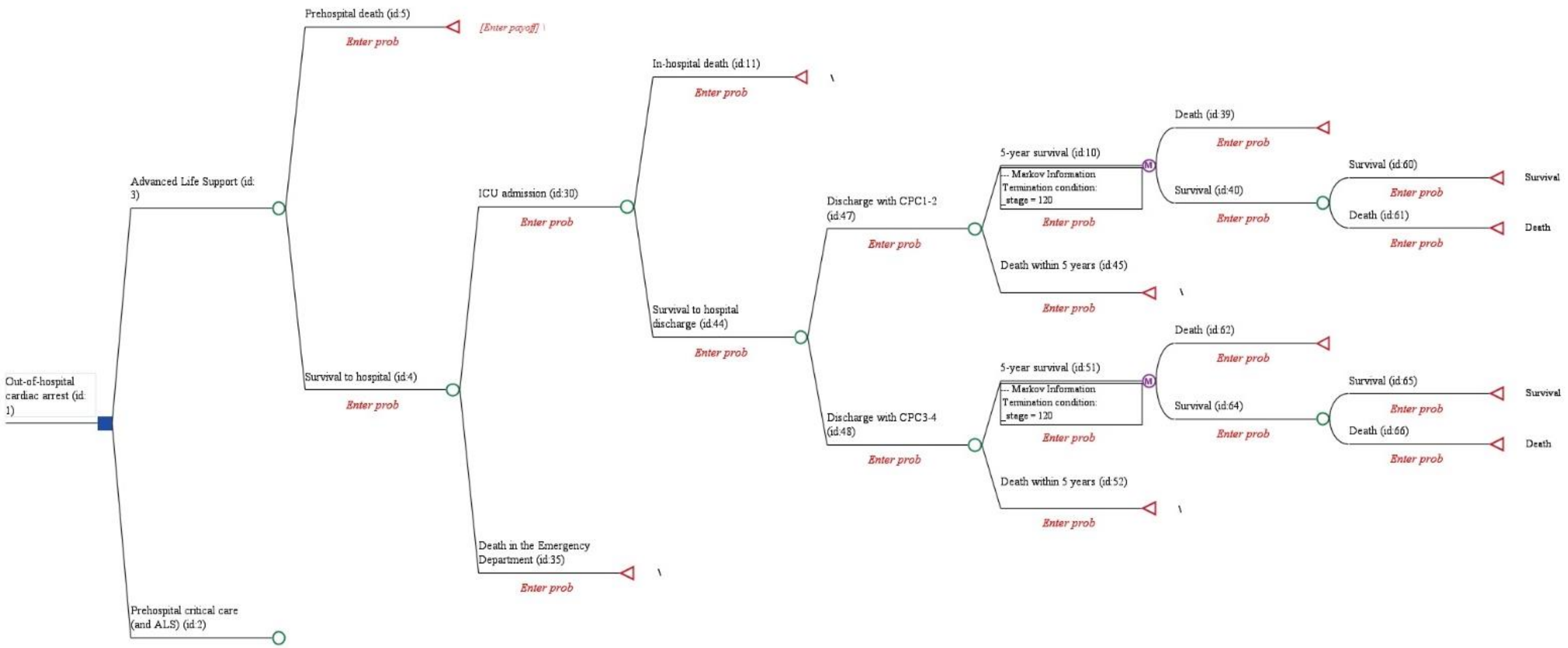

Appendix 2. Search strategy and selection of publications for the synthesis of in-hospital costs of treatment for out-of-hospital cardiac arrest.

PubMed was searched using the search string *(((cost) OR economics) OR expenditure) AND "cardiac arrest"*, with search results limited to the last 10 years. Inclusion criteria were cost analysis of any intervention in adult, non-traumatic OHCA, in the UK. See Figure 1 for a flow chart of the search results.

Figure 1. Flow chart of the selection of publications. Search results from PubMed on 22 November 2017.

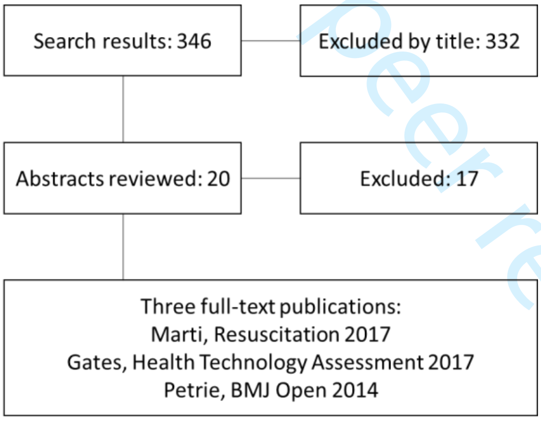

Twelve of the publications for which abstracts were reviewed were excluded as they were undertaken outside the UK, and were therefore expected to have significantly different cost and clinical parameters. The other five excluded studies did not include relevant costs. Of the three full-text publications reviewed, the studies by Marti et al. and Gates et al. related to the same randomised controlled trial of a mechanical chest compression device. Hospital treatment cost estimates were based on the length of stay of patients on the Intensive Care Unit (ICU) and on regular hospital wards as well as Emergency Department (ED) and outpatient costs. In contrast, Petrie et al. included additional significant costs of cardiac interventions, such as percutaneous coronary intervention (PCI), pacemaker insertion or coronary artery bypass graft surgery (CABG), resulting in an overall higher and probably more accurate estimate of hospital costs for OHCA. We therefore used the data from Petrie et al., including the study's open access source data, in combination with the Department of Health NHS Reference Costs 2015-16, for estimating the costs of in-hospital treatment. Post-discharge costs were extracted from Gates et al.

For peer review only

Appendix 3. Search strategy and selection of publications for the synthesis of quality and length of life after discharge from hospital following out-of-hospital cardiac arrest.

Quality and length of life after discharge from hospital following OHCA were estimated from previously published work. We undertook a focused literature search in PubMed, using the search string *(((quality of life[MeSH Terms]) OR utility) OR life expectancy[MeSH Terms]) AND cardiac arrest[MeSH Terms]*, with search results limited to the last 10 years. Inclusion criteria were adult non-traumatic OHCA and outcomes of either length or quality of life measured in utility. Where available, publications using data from the UK were included; where no UK data were available, data from North America or Australia were deemed sufficient. See Figure 1 for a flow chart of the literature review.

Figure 1. Focused literature search for data on length and quality of life following OHCA.

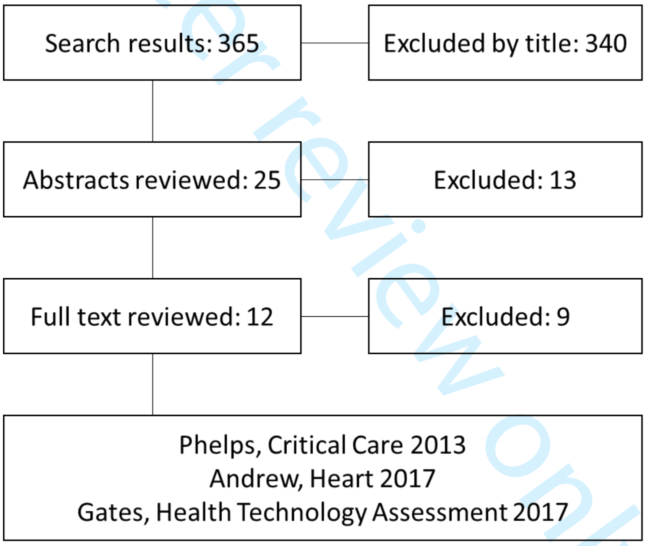

For peer review only

The two questions required for the decision tree and Markov model were

- What is the percentage of Cerebral Performance Category (CPC) 1-2 and CPC 3-4 amongst OHCA patients discharged from hospital?
- What is the quality and length of life of survivors of OHCA with CPC 1-2 and CPC 3-4, respectively?

Percentage of CPC 1-2 and CPC 3-4 at discharge from hospital

Gates et al. provided the most current and relevant information in their randomised controlled trial and embedded economic analysis of mechanical chest compressions during OHCA in the UK. CPC was

measured at three months after discharge from hospital, with 245 of 272 (90%) of patients achieving a CPC 1-2. This is somewhat higher than the 78% and 85% of CPC 1-2 survivors reported by Petrie et al. and Phelps et al. in a UK and Australian patient population, respectively. However, both these studies measured CPC at hospital discharge, rather than at three months after discharge. Patients discharged from hospital after OHCA with CPC 3-4 have a significantly higher mortality in the first months post discharge when compared to those discharged with CPC 1-2. Additional data from Phelps et al. at twelve months post discharge demonstrate that, of the 804 patients surviving to one year post discharge, 90% were discharged from hospital with CPC 1-2. We therefore concluded that 85% was a robust estimate for the percentage of OHCA survivors discharged with CPC 1-2.

Length of life after discharge from hospital

Phelps et al. also provide the most detailed description of long-term survival following OHCA, according to CPC at discharge from hospital, including person-years for the first year and the following four years after hospital discharge. We used these data to model mortality during the first 5 years after discharge from hospital following OHCA. Andrew et al. and Phelps et al. showed that survival rates after 5 years following discharge from hospital after OHCA were the same as those of the standard population. Mortality rates were therefore obtained from the National Life Tables for England and applied to the patient cohort from five years onwards. This resulted in an overall survival rate of 65.0% and 50.0% (CPC1-2) and 36.0% and 27.8% (CPC3-4) at 10 and 15 years post discharge, respectively.

Quality of life after discharge from hospital

In their RCT which recruited a total of 4,471 patients with OHCA between 2010 and 2013 in four UK ambulance services, Gates et al. reported health-related quality of life (HRQL) using the EuroQol – 5 dimension descriptive system (EQ-5D). The EQ-5D has been validated for measuring HRQL and for its use in economic evaluations in a variety of conditions, including cardiac arrest. Gates et al. converted their findings to health-state utilities using the UK tariff. Using a combination of decision tree and Markov model similar to that employed in this research, they calculated a utility 0.75 and 0.47 for the CPC 1-2 and CPC 3-4 groups, respectively. The use of EQ-5D for the calculation of utility is recommended by the National Institute for Health and Care Excellence.

For peer review only

CHEERS checklist—Items to include when reporting economic evaluations of health interventions

Section/item	Item No	Recommendation	Reported on page No/ line No
**Title and abstract**			
Title	1	Identify the study as an economic evaluation or use more specific terms such as “cost-effectiveness analysis”, and describe the interventions compared.	Title, page 1
Abstract	2	Provide a structured summary of objectives, perspective, setting, methods (including study design and inputs), results (including base case and uncertainty analyses), and conclusions.	Page 2
**Introduction**			
Background and objectives	3	Provide an explicit statement of the broader context for the study.	Page 3-4
		Present the study question and its relevance for health policy or practice decisions.	Page 4
**Methods**			
Target population and subgroups	4	Describe characteristics of the base case population and subgroups analysed, including why they were chosen.	Page 5 ‘Clinical setting’
Setting and location	5	State relevant aspects of the system(s) in which the decision(s) need(s) to be made.	Page 5 ‘Clinical setting’
Study perspective	6	Describe the perspective of the study and relate this to the costs being evaluated.	Page 4 ‘Perspective and time horizon’
Comparators	7	Describe the interventions or strategies being compared and state why they were chosen.	Page 5 ‘Alternative interventions...’
Time horizon	8	State the time horizon(s) over which costs and consequences are being evaluated and say why appropriate.	Page 4 ‘Perspective and time horizon’
Discount rate	9	Report the choice of discount rate(s) used for costs and outcomes and say why appropriate.	Page 6 ‘Application of values...’
Choice of health outcomes	10	Describe what outcomes were used as the measure(s) of benefit in the evaluation and their relevance for the type of analysis performed.	Page 5-6 ‘Identification and synthesis...’ Appendix 3
Measurement of effectiveness	11a	*Single study-based estimates:* Describe fully the design features of the single effectiveness study and why the single study was a sufficient source of clinical effectiveness data.	N/A
	11b	*Synthesis-based estimates*: Describe fully the methods used for identification of included studies and synthesis of clinical effectiveness data.	Page 5-6 ‘Identification and synthesis...’ Appendices 2 to 3
Measurement and valuation of preference based outcomes	12	If applicable, describe the population and methods used to elicit preferences for outcomes.	Page 5-6 ‘Identification and synthesis...’ Appendices 2 to 3
Estimating resources and costs	13a	*Single study-based economic evaluation:*Describe approaches used to estimate resource use associated with the alternative interventions. Describe primary or secondary research methods for valuing each resource item in terms of its unit cost. Describe any adjustments made to approximate to opportunity costs.	N/A

For peer review only

Section/item	Item No	Recommendation	Reported on page No/ line No
	13b	*Model-based economic evaluation:* Describe approaches and data sources used to estimate resource use associated with model health states. Describe primary or secondary research methods for valuing each resource item in terms of its unit cost. Describe any adjustments made to approximate to opportunity costs.	Page 5-6 'Identification and synthesis...' Appendices 2 to 3
Currency, price date, and conversion	14	Report the dates of the estimated resource quantities and unit costs. Describe methods for adjusting estimated unit costs to the year of reported costs if necessary. Describe methods for converting costs into a common currency base and the exchange rate.	Page 6 'Application of values...'
Choice of model	15	Describe and give reasons for the specific type of decision-analytical model used. Providing a figure to show model structure is strongly recommended.	Page 5 'Methods' Appendix 1
Assumptions	16	Describe all structural or other assumptions underpinning the decision-analytical model.	Page 5 'Methods' Appendices 1-3 Page 8 'Sensitivity analyses'
Analytical methods	17	Describe all analytical methods supporting the evaluation. This could include methods for dealing with skewed, missing, or censored data; extrapolation methods; methods for pooling data; approaches to validate or make adjustments (such as half cycle corrections) to a model; and methods for handling population heterogeneity and uncertainty.	Page 7, Table 1 Page 8 'Sensitivity analyses'
**Results**			
Study parameters	18	Report the values, ranges, references, and, if used, probability distributions for all parameters. Report reasons or sources for distributions used to represent uncertainty where appropriate. Providing a table to show the input values is strongly recommended.	Table 1
Incremental costs and outcomes	19	For each intervention, report mean values for the main categories of estimated costs and outcomes of interest, as well as mean differences between the comparator groups. If applicable, report incremental cost-effectiveness ratios.	Figure 2, Table , Table 4. We chose median rather than mean, due to the considerably skewed distributions
Characterising uncertainty	20a	*Single study-based economic evaluation* :Describe the effects of sampling uncertainty for the estimated incremental cost and incremental effectiveness parameters, together with the impact of methodological assumptions (such as discount rate, study perspective).	N/A
	20b	*Model-based economic evaluation:* Describe the effects on the results of uncertainty for all input parameters, and uncertainty related to the structure of the model and assumptions.	Page 7, Table 1
Characterising heterogeneity	21	If applicable, report differences in costs, outcomes, or cost-effectiveness that can be explained by variations between subgroups of patients with different baseline characteristics or other observed variability in effects that are not reducible by more information.	Figure 2, Table 3, Table 4
Study findings, limitations, generalisability, and	22	Summarise key study findings and describe how they support the conclusions reached. Discuss limitations	Page 12-14

For peer review only

Section/item	Item No	Recommendation	Reported on page No/ line No
current knowledge		and the generalisability of the findings and how the findings fit with current knowledge.	
**Other**			
Source of funding	23	Describe how the study was funded and the role of the funder in the identification, design, conduct, and reporting of the analysis. Describe other non-monetary sources of support.	Page 3
Conflicts of interest	24	Describe any potential for conflict of interest of study contributors in accordance with journal policy. In the absence of a journal policy, we recommend authors comply with International Committee of Medical Journal Editors recommendations.	Page 3

For consistency, the CHEERS statement checklist format is based on the format of the CONSORT statement checklist

For peer review only

BMJ Open

Cost-effectiveness of Advanced Life Support and prehospital critical care for out-of-hospital cardiac arrest: A decision analysis model

Journal:	*BMJ Open*
Manuscript ID	bmjopen-2018-028574.R1
Article Type:	Research
Date Submitted by the Author:	20-May-2019
Complete List of Authors:	von Vopelius-Feldt, Johannes; The University of the West of England, Faculty of Health and Applied Sciences; University Hospitals Bristol NHS Foundation Trust, Academic Department of Emergency Care<br>Powell, Jane; University of the West of England, Bristol, Centre for Public Health and Wellbeing<br>Benger, Jonathan; The University of the West of England, Faculty of Health and Applied Sciences; University Hospitals Bristol NHS Foundation Trust, Academic Department of Emergency Care
**Primary Subject Heading**:	Emergency medicine
Secondary Subject Heading:	Cardiovascular medicine, Health economics, Intensive care
Keywords:	ACCIDENT & EMERGENCY MEDICINE, Adult intensive & critical care < INTENSIVE & CRITICAL CARE, CARDIOLOGY

SCHOLARONE™
Manuscripts

Cost-effectiveness of Advanced Life Support and prehospital critical care for out-of-hospital cardiac arrest: A decision analysis model

AUTHORS

Johannes von Vopelius-Feldt[1,2] (ORCiD ID 0000-0002-6389-289X)

Jane Powell[3] (ORCiD ID 0000-0002-1828-1184)

Jonathan Benger[1,2] (ORCiD ID 0000-0001-6131-0916)

ASSOCIATIONS

1) Faculty of Health and Applied Sciences, University of the West of England, Bristol, United Kingdom

2) Academic Department of Emergency Care, University Hospitals Bristol NHS Foundation Trust, Bristol, United Kingdom

3) Centre for Public Health and Wellbeing, University of the West of England, Bristol, United Kingdom

CORRESPONDING AUTHOR

Johannes von Vopelius-Feldt

Emergency Care Research Group

1H14, Glenside Campus

Blackberry Hill, BS16 1DD Bristol

University of the West of England, Bristol

johannes.vonvopelius-feldt@uwe.ac.uk

Phone: 0044 (0)7958064069

WORD COUNT

3,141

For peer review only - http://bmjopen.bmj.com/site/about/guidelines.xhtml

ABSTRACT

Objectives

This research aimed to answer the following questions: What are the costs of prehospital Advanced Life Support (ALS) and prehospital critical care for out-of-hospital cardiac arrest (OHCA)? What is the cost-effectiveness of prehospital ALS? What improvement in survival rates from OHCA would prehospital critical care need to achieve in order to be cost-effective?

Setting

A single National Health Service (NHS) ambulance service and a charity funded prehospital critical care service.

Participants

The patient population is adult, non-traumatic OHCA.

Methods

We combined data from previously published research with data provided by a regional ambulance service and air ambulance charity to create a decision tree model, coupled with a Markov model, of costs and outcomes following OHCA. We compared no treatment for OHCA to the current standard of care of prehospital ALS, and prehospital ALS to prehospital critical care. To reflect the uncertainty in the underlying data, we used probabilistic and two-way sensitivity analyses.

Results

Costs of prehospital ALS and prehospital critical care were £347 and £1,711 per patient, respectively. When costs and outcomes of prehospital, in-hospital and post-discharge phase of OHCA care were combined, prehospital ALS was estimated to be cost-effective at £11,407 per quality-adjusted life year (QALY). In order to be cost-effective in addition to ALS, prehospital critical care for OHCA would need to achieve a minimally economically important difference (MEID) in survival to hospital discharge of 3-5%.

Conclusions

This is the first economic analysis to address the question of cost-effectiveness of prehospital critical care following OHCA. While costs of either prehospital ALS and/or critical care per patient with OHCA are relatively low, significant costs incur during hospital treatment and after discharge in patients who survive. Knowledge of the MEID for prehospital critical care can guide future research in this field.

Trial registration

ISRCTN registry ID ISRCTN18375201

For peer review only - http://bmjopen.bmj.com/site/about/guidelines.xhtml

STRENGTHS AND LIMITATIONS OF THIS STUDY

- This is the first study to describe the cost-effectiveness of the complete care pathway for out-of-hospital cardiac arrest in England
- This study uses a combination of primary and secondary data, therefore optimising the use of currently available evidence
- The decision analysis model includes a combination of probabilistic and two-way sensitivity analyses to address the complex question of cost-effectiveness of prehospital critical care in a transparent manner
- A limitation of this study is that is based on theoretic modelling only; the uncertainty in the underlying data is addressed by the methods and reflected in the presentation of results

FUNDING

This work is funded by a National Institute for Health Research (NIHR) doctoral research fellowship for Johannes von Vopelius-Feldt (DRF-2015-08-040). The funder is not involved in the design of the study or collection, analysis and interpretation of data, or in writing the manuscript. The views expressed are those of the author(s) and not necessarily those of the NHS, the NIHR or the Department of Health.

COMPETING INTEREST

JVVF and JB work as prehospital doctors with a regional prehospital critical care team.

INTRODUCTION

Rates of survival following out-of-hospital cardiac arrest (OHCA) remain low in the UK, with an overall survival rate to hospital discharge of 7.9%.[1] One area of potential improvement is the provision of prehospital care by the Emergency Medical Services (EMS).[2] The current standard of prehospital care in the UK is the provision of Advanced Life Support (ALS) by appropriately trained paramedics.[3] In an attempt to improve survival rates following OHCA, prehospital critical care teams (CCTs) are dispatched to OHCA in some regions in the UK, in addition to the standard ALS response.[4, 5] CCTs consist of specialist paramedics and/or doctors, with extended skills and competences, which we previously described in detail.[6] They are frequently based on air ambulances, with funding received variably through charitable donations and/or the National Health Service (NHS).[7]

For peer review only - http://bmjopen.bmj.com/site/about/guidelines.xhtml

The cost-effectiveness of ALS care provided by paramedics or physicians has been studied previously.[8, 9] However, generalisability of this evidence to current UK practice is limited due to the time elapse since the research was undertaken and considerable differences in EMS configurations. The cost-effectiveness of prehospital critical care for OHCA has not been researched in sufficient detail to guide funding decisions. A recently published systematic review of the effectiveness of CCTs for OHCA showed a potential small to moderate survival benefit but also identified issues limiting internal and external validity of the included observational studies.[10]

Economic analyses have been undertaken in regards to the cost-effectiveness of helicopter-based EMS care (HEMS) for trauma, with mixed results.[11, 12] To our knowledge, no economic analysis exists which addresses the question of cost-effectiveness of prehospital critical care for OHCA. In addition, a recent study of stakeholders' views of prehospital care for OHCA revealed contrasting opinion of whether current NHS funding of prehospital ALS care for OHCA represents an appropriate distribution of limited healthcare resources in the UK.[13] Adding to the complexity of cost-effectiveness of prehospital ALS or critical care for OHCA is the fact that the majority of costs occur along the patient care pathway in hospital or post-discharge.[14, 15]

In the absence of robust data regarding the effectiveness of prehospital ALS or critical care for OHCA in the UK setting, this study addresses the following questions

- What are the costs of prehospital ALS and prehospital care for OHCA in the UK?
- What is the cost-effectiveness of the current OHCA treatment pathway from prehospital ALS care to post-discharge costs, when compared to no treatment?
- What improvement in survival rates from OHCA would prehospital critical care need to achieve in order to be cost-effective, when compared to ALS?

METHODS

We combined data from previously published research with costs provided by a regional EMS provider and air ambulance charity to create a decision analysis model.

Development of the decision analysis model

The initial decision analysis model for this research considered three potential outcomes: prehospital death, in-hospital death or survival to hospital discharge. After a focused review of the literature, it became clear that differences in in-hospital treatment costs and survival with either poor or favourable neurological outcome have considerable impact on the cost-effectiveness estimates.[14, 15] We therefore expanded the model to include these important branches in a combination of

For peer review only - http://bmjopen.bmj.com/site/about/guidelines.xhtml

decision tree model for prehospital and hospital costs and outcomes, and a Markov model for post-discharge costs and outcomes. The structure is similar to the model used by Gates et al.[15] in their economic analysis of mechanical chest compression during prehospital care for OHCA. Figure 1 shows the final model (for simplicity, only the ALS arm of the model is displayed). Important assumptions, based on literature referenced in the sections below, are that

- A proportion of patients survive to hospital arrival but only receive palliative care, resulting in death in the emergency department or shortly afterwards.
- Patients who survive to hospital discharge with cerebral performance category (CPC) of 3-4 have a higher rate of survival following the first 5 years after hospital discharge, compared to those patients surviving with CPC 1-2.
- After 5 years post-discharge, annual survival rates for patients still alive at this point are the same as those of the average population.

Perspective and time horizon

In keeping with the recommendations of the National Institute for Health and Care Excellence (NICE), the perspective on costs chosen for this economic evaluation was that of the National Health Service (NHS) and personal social services.[16] The perspective on outcomes included all direct health effects on patients, namely length and quality of life (quality-adjusted life years, QALYs) with a corresponding long-term time horizon.

Clinical setting

The economic analysis was undertaken in the section of an NHS ambulance trust in England which was covered by a critical care team. It covers an area of 2.930 square miles with a population of approximately 2.5 million people. The area is largely suburban or rural with one metropolitan area and two urban areas. The overall population density is 860/square mile. The patient population is adult, non-traumatic OHCA.

Alternative interventions – Advanced life support and prehospital critical care

The NHS ambulance trust provided ALS paramedic care, using a combination of first responders, rapid response vehicles and ambulance. The section of the ambulance trust included in this analysis contained 27 ambulance stations with a total of approximately 800 ALS paramedics (employed part- or full-time) as well as emergency care assistants (ECAs) and ambulance technicians (data provided by ambulance trust). The median response time for OHCA during the study period was 7.4min (IQR 4.9min – 11.4min) and care on scene was provided by a mean of 2.43 core resources (paramedic-staffed double-crewed ambulances and/or rapid response vehicles). The prehospital critical care service was funded largely through an air ambulance charity but also received support from the

For peer review only - http://bmjopen.bmj.com/site/about/guidelines.xhtml

ambulance trust. It provided prehospital critical care cover from 7.00am to 1.00am, with access to a helicopter and rapid response vehicles. The team consisted of specialist paramedics in critical care and prehospital critical care doctors. The service responded to OHCAs in addition to the usual ambulance trust's ALS response.

Identification and synthesis of evidence

Costs

Financial data was obtained from the ambulance trust and air ambulance charity for the financial year 2015/16. This included pay for clinical and non-clinical staff as well as costs of dispatch infrastructure, ambulance stations, vehicles, fuel, medical supplies, communications equipment, community first responders and 3rd party costs. The amount of funding spent on OHCA was calculated as the number of OHCA cases divided by the number of cases of any aetiology. Calculating the proportion of resources spent on OHCA in this fashion distributes the costs of waiting time of resources in between emergency proportionally between OHCA cases and those of other aetiology.[17] The costs of in-hospital and post-discharge treatment were synthesised from relevant recent publications, identified by a focused systematic search of the literature (appendix 1).[14, 15, 18]

Outcomes

After a literature review (appendix 2), the following outcomes of interest were included in the decision analysis model (figure 1): survival to arrival at hospital; survival to ICU admission; survival to hospital discharge; CPC at discharge from hospital; 5-year survival following hospital discharge; annual mortality and quality of life. Prehospital outcomes after OHCA with ALS care were based on a one-year data sample provided by the participating ambulance trust (2016), while in-hospital outcomes were based on recent literature of OHCA in the UK.[19-21] For prehospital critical care, a range of effect sizes were simulated in two-way sensitivity analyses.[10] This approach was chosen to transparently reflect the considerable uncertainty of the effectiveness of prehospital critical care for OHCA in current research.[10] Post-discharge outcomes for the first five years after hospital discharge were retrieved from publications identified through a further focused systematic search of the literature (appendix 2).[15, 22] Further long-term outcomes (quality of life) were modelled according to survival rates provided by the UK's Office for National Statistics.[23, 24]

Application of values and distributions to the model

Prior to inclusion in the model, all costs were adjusted to Pound Sterling for the financial year 2016-17 according to the UK Hospital and Community Health Services (HCHS) pay and prices index.[25] In keeping with NICE guidelines, an annual discount rate of 3.5% was applied to costs and effects which occurred after the first year following discharge from hospital.[16] Table 1 provides a comprehensive

For peer review only - http://bmjopen.bmj.com/site/about/guidelines.xhtml

list of all model parameters, based on the sources of data described in the previous section. The confidence intervals or interquartile ranges of the data were transferred to sampling distributions.[26] In keeping with previous recommendations, we used Gamma distributions for costs and Beta distributions for probabilities.[26] Each parameter in table 1 corresponds to either a cost, effect or probability of each chance node or Markov node of the decision analysis model in figure 1. For costs and effects, these are simply added up for each potential outcome. For example, in-hospital death after prehospital critical care would incur the costs of prehospital critical care plus ALS, costs of ED treatment, and ICU and non-ICU costs of non-survivors. The effect is 0 QALYs, as the outcome is death. The probabilities for each node were entered into the model as the values described in table 1, with the exception of the probability of survival to hospital discharge. To create a 10% rate of survival to hospital discharge after prehospital critical care, for example, the probability value for the corresponding node would need to be entered as a function of '10%, dividided by *p ICU admission*, divided by *p survival to hospital'*.

For peer review only

For peer review only - http://bmjopen.bmj.com/site/about/guidelines.xhtml

Table 1. Values and distributions of parameters of the decision tree and Markov model; all costs adjusted for inflation to the financial year 2016-17

**Parameters**	**Cohort / patient group**	**Mean value (95% confidence interval)**	**Distribution**	**Source**
**Prehospital (decision tree model)**				
Costs of prehospital care	ALS	£347	Gamma	Own data
	Critical care (in addition to ALS)	£2,058 (£1,711 critical care plus £347 ALS care)	N/A	
p survival to hospital	ALS	0.26 (0.25 – 0.27)	Beta	von Vopelius-Feldt [10]
	Critical care	0.25 – 0.31	N/A	
**In-hospital (decision tree model)**				
Costs of ED treatment		£377 (£355 – £399)	Gamma	Department of Health [27]
p ICU admission*		0.65 (0.58 – 0.71)	Beta	Benger [19]
Costs of ICU treatment (daily) and length of stay on ICU (LOS)	Survival to hospital discharge	£1,745 (£1,654 – £1,836)<br>LOS (days) 5.7 (5.4 – 6.0)	Gamma	Petrie [14]<br>Nolan [20]
	In-hospital death	£1,768 (£1,668 – £1,868)<br>LOS (days) 2.4 (2.3 – 2.5)		
Cost of non-ICU treatment (total)	Survival to hospital discharge	£12,823 (£10,123 – £15,523)	Gamma	Petrie [14]<br>Nolan [20]
	In-hospital death	£3,835 (£3,045 – £4,625)		
p survival to hospital discharge**	ALS	0.09 (0.08 – 0.10)	Beta	von Vopelius-Feldt [10]
	Critical care	0.10 – 0.15	N/A	
**Post-discharge (decision tree model)**				
p survival with CPC1-2***		0.85 (0.826 – 0.871)	Beta	Phelps [22]
p 5-year survival	CPC1-2	0.740 (0.709 – 0.768)	Beta	Phelps [22]
	CPC3-4	0.408 (0.332 – 0.489)		
Person-years per death within 5 years	CPC1-2	1.827 (1.750 – 1.878)	Gamma	Phelps [22]
	CPC3-4	0.313 (0.254 – 0.375)		
Utility	CPC1-2	0.75 (0.70 – 0.80)	Beta	Gates [15]
	CPC3-4	0.47 (0.42 – 0.52)		
Annual healthcare costs	CPC1-2	£3,358	Gamma	Gates [15]
	CPC3-4	£43,670		
**Long-term outcomes (Markov model)**				
p annual mortality after 5 years post discharge	CPC1-2	National age-adjusted mortality rates	Exact values	Office for National Statistics [23], Andrew [24]
	CPC3-4			

*of survivors to hospital **of all cases of OHCA ***of survivors to hospital discharge
ALS: Advanced Life Support, N/A: not applicable, p: probability, ED: emergency department, ICU: intensive care unit, LOS: length of stay, CPC: Cerebral Performance Category

For peer review only

For peer review only - http://bmjopen.bmj.com/site/about/guidelines.xhtml

Sensitivity analyses

As the analysis was based largely on secondary data, we undertook a probabilistic sensitivity analysis of the decision analysis model, to reflect the underlying uncertainty in the data.[26] We ran 1,000 iterations of the decision analysis model in a Monte Carlo simulation. For each iteration, the values of all model parameters were drawn at random from the sampling distributions described in table 1. In addition, the probabilities of survival to hospital admission and survival to hospital discharge after prehospital critical care were subject to a two-way sensitivity analysis, reflecting a range of potential effects based on a recent systematic review.[10] All analysis was undertaken in TreeAge (Version 2017).

Model validation

Validity of decision analysis models can be examined in a number of different ways.[26] Face validity was examined by presenting the model, values and results to a group of prehospital clinicians and air ambulance charity staff. Internal validity was assessed through the sensitivity analyses described above, as well as selecting extreme values for each individual parameter in the model and checking if the results were influenced as expected. We also undertook visual inspection of the values selected by the software for each variable during the probabilistic sensitivity, to assure that values were in the ranges expected for the parameter. While the software produced an error message if probabilities either did not add up to 1 or had individual values of less than 0 or more than 1, we also hand checked all probabilities to assure that the overall probability of death / survival was correctly calculated from the probabilities at each step of the decision tree model.

Presentation of results

In keeping with the three research questions outlined in the introduction, the results will be presented in three sections. First, we will describe the costs occurring during prehospital care for OHCA, for both ALS and critical care. Outcome measure will be Pound Sterling (£). We will then describe the cost-effectiveness of prehospital ALS, including the complete patient pathway, when compared to no treatment. The outcome measure will be the incremental cost-effectiveness in £/QALY. Finally, we will analyse the MEID which prehospital critical care would need to achieve in order to be cost effective at a willingness-to-pay (WTP) threshold of £20,000/QALY. The outcome measure will be the absolute difference in survival to hospital discharge between ALS and prehospital critical care which fulfils the MEID criteria.

Patient and public involvement

A Patient and Public Involvement (PPI) Group was consulted during the application and planning process of the doctoral research fellowship which underlies this research. In addition, the PPI group's

For peer review only - http://bmjopen.bmj.com/site/about/guidelines.xhtml

as well as other relevant stakeholders' views expressed during previous research[13] informed the methods of this research. Results will be disseminated to all stakeholders who participated, through an executive summary of this research.

Ethics and consent

With the exception of the financial data of prehospital care costs, all data were obtained from previously published research or government documents. Therefore, patient consent was not required. The research was reviewed and approved by the Sheffield National Research Ethics Committee, York and Humber on 29 July 2016, reference number 16/YH/0300.

RESULTS

Prehospital costs

Table 2 gives an overview of the costs of prehospital ALS care and prehospital critical care for OHCA. The total expenditure for ALS care is over 25 times higher than the costs of providing prehospital critical care for the same geographical area. However, OHCAs represent approximately one third of the work load of the prehospital critical care team, whereas only 0.85% of ALS care funding can be attributed to OHCA care. The average cost of a patient with OHCA being attended by ALS paramedics is £347, while the cost for attendance of a prehospital critical care team is £1,711 (plus £347, as ALS resources would still be dispatched with critical care team attendance).

For peer review only

For peer review only - http://bmjopen.bmj.com/site/about/guidelines.xhtml

Table 2. Prehospital treatment costs for out-of-hospital cardiac arrest

	ALS care	Critical care
**Total expenditure 2015-16 (in £)**		
Staff costs	36,732,844	665,293
Vehicles (including fuel)	8,145,245	13,659
Helicopter (including fuel)	-	1,318,432
Buildings	2,705,442	37,693
Equipment	1,681,383	63,296
Other	6,261,901	37,752
Dispatch centre	2,226,955	45,000
**Total**	**57,753,770**	**2,181,125**
**Expenditure per OHCA 2015-16 (in £)**		
**Percentage of resources devoted to OHCA**	0.85%	30%
**Number of OHCA patients**	1412	388
**Cost per OHCA patient**	**347**	**1,711**

Cost-effectiveness of Advanced Life Support for OHCA

The estimated cost-effectiveness of the current standard pathway for OHCA, including prehospital ALS, hospital admission, ICU and non-ICU treatment, as well as post-discharge healthcare costs, is £11,407/QALY (median, IQR £6,840 /QALY – £16,863/QALY), when compared to no treatment. Figure 2 shows the corresponding scatterplot with results from 1,000 iterations of the probabilistic sensitivity analysis. As the majority of these 1,000 results lie below the WTP threshold of £20,000/QALY (approximately 93%), prehospital ALS for OHCA can be assumed to be cost-effective at this WTP threshold with a high degree of certainty.

Incremental cost-effectiveness of prehospital critical care for OHCA

Figure 3 shows the cost-effectiveness acceptability curves for a number of potential treatment effects of prehospital critical care following OHCA. For this model, the ratio of rates of survival to arrival at hospital and survival to hospital discharge in the prehospital critical care treatment arm was kept constant. Rates of survival to hospital arrival and of survival to hospital discharge after ALS care for OHCA were 25.8% and 9.0%, respectively. The intersections of the WTP threshold and the cost-acceptability curves provide the probability of prehospital critical care being cost-effective. Prehospital critical care needs to achieve a 2% or 3% absolute increase in survival to hospital discharge to have a 48% or 72% probability of being cost effective at a WTP threshold of £20,000 per QALY, respectively. With a lower absolute difference in survival rates of 1%, prehospital critical care has a less than 1% probability of being cost effective at the same WTP threshold. Higher absolute differences

For peer review only - http://bmjopen.bmj.com/site/about/guidelines.xhtml

in survival of 4%, 5% and 6% result in probabilities of cost-effectiveness at the WTP threshold of £20,000 of 84%, 90% and 93%, respectively.

The results of the analysis presented in figure 3 assume that the rates of survival to hospital increase proportionally with the rates of survival to hospital discharge. However, as the majority of costs for the care of OHCA is generated by in-hospital treatments, variations in the rate of survival to hospital can have considerable influence on costs, independently of later survival to hospital discharge. Table 3 provides estimates and interquartile ranges for possible combinations of survival to hospital and survival to hospital discharge rates. As table 3 demonstrates, higher rates of survival to hospital arrival result in moderate increases in incremental costs of prehospital critical care for OHCA.

Table 3. Incremental cost-effectiveness of prehospital critical care for OHCA (in £ per quality-adjusted life year), for different rates of survival to hospital and survival to hospital discharge, compared to ALS.

**Difference in rates of survival to hospital**	**Difference in rates of survival to hospital discharge (median, 95% CI)**					
	**+1%**	**+2%**	**+3%**	**+4%**	**+5%**	**+6%**
**-5%**	25,000 (16,500 – 46,000)	18,000 (12,500 – 28,500)	15,500 (11,000 – 23,500)	14,500 (10,500 – 21,000)	Not achievable	Not achievable
**0%**	**27,500 (20,000 – 47,500)**	19,000 (14,500 – 30,000)	16,500 (12,500 – 24,500)	15,000 (11,500 – 22,000)	14,000 (11,000 – 20,000)	13,500 (10,500 – 19,000)
**+5%**	30,500 (21,500 – 53,000)	**20,500 (15,500 – 33,500)**	17,000 (13,000 – 26,500)	15,500 (12,000 – 23,000)	14,500 (11,500 – 21,500)	14,000 (11,000 – 20,000)
**+10%**	33,500 (22,500 – 61,000)	22,000 (16,000 – 37,500)	**18,000 (14,000 – 28,500)**	**16,500 (12,500 – 24,500)**	15,000 (12,000 – 22,000)	14,500 (11,500 – 21,000)
**+15%**	36,500 (23,000 – 70,500)	23,500 (17,000 – 42,000)	20,000 (14,500 – 32,000)	17,000 (13,000 – 26,500)	**16,000 (12,500 – 23,500)**	**15,000 (11,500 – 22,000)**
**+20%**	39,500 (23,500 – 79,500)	24,500 (17,000 – 46,000)	20,000 (15,000 – 34,000)	17,500 (13,500 – 29,000)	16,500 (12,500 – 25,500)	15,500 (12,000 – 23,000)

Light and darker shades of grey indicate most likely combination of differences in rates of survival to hospital and survival to hospital discharge. All costs are in £ and have been rounded to the nearest £500 value.

Table 4 presents a further sensitivity analysis of the effects of varying costs of prehospital critical care and rates of survival to hospital discharge. Variations in the costs of prehospital critical care for OHCA only have a minor to moderate effect on its cost-effectiveness.

For peer review only - http://bmjopen.bmj.com/site/about/guidelines.xhtml

Table 4. Incremental cost-effectiveness of prehospital critical care for OHCA (in £ per quality-adjusted life year), for a range of costs of prehospital critical care and different rates of survival to hospital discharge, compared to ALS.

Difference in costs of prehospital care	Difference in rates of survival to hospital discharge (median, 95% CI)					
	**+1%**	**+2%**	**+3%**	**+4%**	**+5%**	**+6%**
**-50%**	20,000 (16,000 – 33,500)	16,000 (13,000 – 25,500)	15,000 (12,000 – 23,000)	14,000 (11,500 – 21,500)	14,000 (11,000 – 21,000)	13,500 (11,000 – 20,500)
**-20%**	25,000 (19,000 – 43,000)	18,500 (14,500 – 30,500)	16,500 (13,000 – 26,000)	15,500 (12,500 – 24,000)	15,000 (12,000 – 23,000)	14,500 (11,500 – 22,000)
**0%**	**28,000 (21,500 – 50,500)**	**20,000 (16,000 – 33,500)**	**17,500 (14,000 – 28,500)**	**16,000 (13,000 – 25,500)**	**15,500 (12,500 – 24,000)**	**15,000 (12,000 – 23,000)**
**+20%**	31,500 (24,000 – 57,500)	22,000 (17,000 – 37,000)	18,500 (14,500 – 30,500)	17,000 (13,500 – 27,000)	16,000 (13,000 – 25,500)	15,500 (12,500 – 24,000)
**+50%**	36,000 (27,500 – 67,500)	24,00 (18,500 – 41,500)	20,000 (16,000 – 33,500)	18,000 (14,500 – 30,000)	17,000 (13,500 – 27,000)	16,000 (13,000 – 25,500)

All costs are in £ and have been rounded to the nearest £500 value.

DISCUSSION

In this decision model analysis, the cost-effectiveness of prehospital ALS care or prehospital critical care for OHCA was determined to a considerable degree by their effects on short and long-term survival, with the majority of costs incurring during hospital treatment or post-discharge.

Prehospital costs vs later costs

Given the complexity of the economic analysis of the whole OHCA pathway, it is worth first looking at prehospital resource use only. The ambulance service described in this analysis had an annual spending of over 50 million pounds sterling. However, during the study period, patients with OHCA only represented 0.22% of all emergency calls attended. Even with the requirement for more resources per OHCA patient compared to most other prehospital conditions, only 0.82% of the annual spending was allocated to OHCAs, at an estimated cost of £347 per OHCA. This might seem a rather low amount of money to be spent on the immediate care for a life or death situation.[13]

For peer review only - http://bmjopen.bmj.com/site/about/guidelines.xhtml

In contrast, the annual costs of providing a helicopter-based prehospital critical care team, covering the same geographic area was relatively low, at just over 2 million pounds per year. Due to the fact that the critical care team attended much fewer patients (approximately 1,200 during the study period) with a higher proportion of OHCAs (30%), the incremental costs of prehospital critical care per OHCA were estimated at £1,711. Given the gravity of the acute situation of an OHCA, this incremental cost might be considered an effective use of EMS resources.

Focusing only on prehospital costs of OHCA would make comparison to other healthcare interventions difficult, and would ignore the importance of costs accumulating further along the OHCA pathway. For patients with OHCA, where resuscitation is unsuccessful and who are declared dead on scene, no further costs occur. However, a large proportion (25.8% in this cohort) of patients with OHCA survive the prehospital phase of their care and are admitted to a hospital. We estimated the costs of in-hospital treatment to be approximately £22,000 for patients surviving to hospital discharge and £8,500 for patients who die in hospital.[14, 20] The major contributors to these costs are ICU-bed days (all patients) and interventions such as PPCI, ICD implantation or coronary artery bypass graft surgery (almost exclusively in survivors).[14] Further costs can accumulate after hospital discharge, most importantly the long-term care services required for the small proportion of patients who survive to hospital discharge with poor neurological function (over £40,000 per year).[15]

Cost-effectiveness of Advanced Life Support for OHCA

We estimated the cost-effectiveness of paramedic-delivered ALS for OHCA to be approximately £11,500 per QALY. With the upper limit of the interquartile range at approximately £16,800, this makes ALS for OHCA almost certainly cost-effective at a NICE WTP threshold of £20,000 per QALY.[16]

While a number of previous publications address the cost-effectiveness of individual aspects of prehospital care or hospital care for OHCA,[28-30] only few address the cost-effectiveness of ALS for OHCA in the context of the complete patient pathway. Naess et al.[31] estimated the cost-effectiveness of ALS for OHCA in Norway to be approximately £6,000 per QALY in 2004. This much lower estimate is likely due to inflation since the year of publication but also development of in-hospital cardiac arrest care with higher rates of interventions in modern OHCA care, and the costs associated with this.[20, 32] More recently, Ginsberg et al.[33] reported the cost-effectiveness of ALS for OHCA to be $28,864 per Disability Adjusted Life Year (DALY) averted, in an Israeli EMS, corresponding to approximately £15,000 - £28,500 per QALY.[34] Despite some uncertainty of the data underlying this analysis, and taking previous research findings into account, we can be fairly certain that paramedic-delivered ALS is a cost-effective treatment for OHCA in the UK. In addition, In

For peer review only - http://bmjopen.bmj.com/site/about/guidelines.xhtml

terms of £ per QALY, it compares favourably with a range of interventions currently funded by the NHS.[35, 36]

Costs of prehospital critical care

As the current observational research describing the effectiveness of prehospital critical care for OHCA is inconsistent and likely suffers from confounding,[10] we decided not to include it in this probabilistic sensitivity analysis. Instead, we used a range of potential absolute effect sizes on rates of survival to hospital discharge, ranging from 1% to 6%, reflecting the most likely range of effect sizes.[10] This sensitivity analysis also allowed us to determine a minimally economically important difference (MEID) in survival rates which would need to be achieved by prehospital critical care services in order to be cost-effective.

At a WTP threshold of £20,000 per QALY, the MEID is in the range of 3-5% absolute improvement in survival to hospital discharge after OHCA. Any improvement in survival rates after OHCA of less than 3%, caused by prehospital critical care services, is very unlikely to be considered cost-effective within an NHS setting.[16] The exact value for the MEID depends on how certain stakeholders want to be about the cost-effectiveness estimate at a given WTP (figure 3), the rates of survival to hospital admission (table 3) and the exact costs of a given prehospital critical care service (table 4). The MEID can be used to guide sample size calculations of future research and to frame the expectations or aims of stakeholders and prehospital critical care services providing care for OHCA.[13] Interestingly, the MEID of prehospital critical care for OHCA calculated in this analysis equals the minimally clinically important difference (MCID) in survival following OHCA described in previous research.[37]

LIMITATIONS

This economic analysis is based on a theoretical construct of the costs and effects of the care pathway for OHCA. As such, assumptions about events occurring in reality had to be made, and have to be assumed to reflect reality accurately, in order for the model to be internally valid.[26] On the other hand, using a modelling approach allowed us to incorporate a considerable number of data sources and to undertake a range of sensitivity analyses, which improves generalisability. While we undertook steps to assure face validity and internal validity of the model, we are unable to reliable test external validity (through simulation of real life events).

We did not adapt a micro-costing approach which would have, for example, included the exact number of paramedics at scene for an OHCA or the amount of drugs or equipment used. Such an approach would have likely resulted in an overall more accurate estimate of the costs of ALS care for

For peer review only - http://bmjopen.bmj.com/site/about/guidelines.xhtml

OHCA, but would have required tremendous efforts to collect and analyse the data. As costs occurring in the later stages of care for OHCA were much higher than during the prehospital phase, a micro-costing approach would have been unlikely to change the cost estimate for prehospital care significantly. Reassuringly, the estimated cost of £347 for ALS care for OHCA compares realistically to the NHS National Schedule of Reference Costs of an average of £236 for *'see and treat and convey'* ambulance activities (considering the higher resource use for OHCA patients).[27]

The main driver of costs for prehospital critical care was the helicopter, followed by staffing costs. We are aware of a number of prehospital critical care services which attend OHCAs using only ground vehicles. This would certainly reduce overall costs of the service (and is therefore reflected in the sensitivity analysis) but also the number of OHCAs which the CCT could reach, particular in rural/suburban areas. In regards to the validity of the overall model, there are few previous publications to compare to. Reassuringly, Delgado et al.[38] used a similar decision model analysis approach to examine the potential cost-effectiveness of HEMS for trauma cases in the US. They found that a 3.7% absolute decrease in mortality was required for HEMS to be cost-effective at a WTP of $50,000 (£38,000) per QALY.

This analysis focuses exclusively on adult, non-traumatic OHCA, as OHCA due to trauma or occurring in the paediatric population is much rarer and due to distinctively different pathology. Results should not be extrapolated to these situations.

CONCLUSION

This is the first economic analysis, based on a decision analysis model, to address the question of cost-effectiveness of prehospital critical care following OHCA. While costs of either prehospital ALS and/or critical care per patient with OHCA are relatively low, significant costs incur during hospital treatment and after discharge in patients who survive. Taking the whole OHCA care pathway into account, prehospital ALS as currently delivered in most NHS ambulance trusts is very likely to be cost-effective at a WTP of £20,000 per QALY. For prehospital critical care to be cost-effective at the same WTP threshold, it would need to improve survival from OHCA by approximately 3-5%.

For peer review only - http://bmjopen.bmj.com/site/about/guidelines.xhtml

REFERENCES

1. Hawkes C, Booth S, Ji C, et al. Epidemiology and outcomes from out-of-hospital cardiac arrests in England. *Resuscitation* 2017;110:133-140.
2. Boyce LW, Vliet Vlieland TP, Bosch J, et al. High survival rate of 43% in out-of-hospital cardiac arrest patients in an optimised chain of survival. *Neth Heart J* 2015;23(1):20-5.
3. Brown S, Kumar D, Millins M, et al. UK ambulance services clinical practice guidelines 2016. Bridgewater: Class Professional 2016.
4. von Vopelius-Feldt J, Benger J. Critical care paramedics in England: a national survey of ambulance services. *Eur J Emerg Med* 2014;21(4):301-4.
5. Hyde P, Mackenzie R, Ng G, et al. Availability and utilisation of physician-based pre-hospital critical care support to the NHS ambulance service in England, Wales and Northern Ireland. *Emerg Med J*, 2012;29(3):177-181.
6. von Vopelius-Feldt, J. and J. Benger, Who does what in prehospital critical care? An analysis of competencies of paramedics, critical care paramedics and prehospital physicians. *Emerg Med J* 2014;31(12):1009-13.
7. Association of Air Ambulances Annual Report 2016. Henley in Arden: Association of Air Ambulances 2017.
8. Nichol G, Laupacis A, Stiell IG, et al. Cost-effectiveness analysis of potential improvements to emergency medical services for victims of out-of-hospital cardiac arrest. *Ann Emerg Med* 1996;27(6):711-20.
9. Yen ZS, Chen YT, Ko PC, et al. Cost-effectiveness of different advanced life support providers for victims of out-of-hospital cardiac arrests. *J Formos Med Assoc* 2006;105(12):1001-7.
10. von Vopelius-Feldt J, Brandling J, Benger J. Systematic review of the effectiveness of prehospital critical care following out-of-hospital cardiac arrest. *Resuscitation* 2017;114:40-46.
11. Taylor CB, Stevenson M, Jan S, et al. A systematic review of the costs and benefits of helicopter emergency medical services. *Injury* 2010;41(1):10-20.
12. Snooks HA, Nicholl JP, Brazier JE, et al. The costs and benefits of helicopter emergency ambulance services in England and Wales. *J Public Health Med* 1996;18(1):67-77.

For peer review only - http://bmjopen.bmj.com/site/about/guidelines.xhtml

13. Von Vopelius-Feldt J, Brandling J, Benger J. Variations in stakeholders' priorities and views onrandomisation and funding decisions in out-of-hospital cardiacarrest: An exploratory study. *Health Sci Rep* 2018;1(9):e78.

14. Petrie J, Easton S, Naik V, et al. Hospital costs of out-of-hospital cardiac arrest patients treated in intensive care; a single centre evaluation using the national tariff-based system. *BMJ Open* 2015;5(4):e005797.

15. Gates S, Lall R, Quinn T, et al. Prehospital randomised assessment of a mechanical compression device in out-of-hospital cardiac arrest (PARAMEDIC): a pragmatic, cluster randomised trial and economic evaluation. *Health Technol Assess* 2017;21(11):1-176.

16. National Institute for Health and Care Excellence. Guide to the methods of technology appraisal 2013. www.nice.org.uk/process/pmg9 (accessed 14 December 2018).

17. Lerner EB, Garrison HG, Nichol G, et al. An Economic Toolkit for Identifying the Cost of Emergency Medical Services (EMS) Systems: Detailed Methodology of the EMS Cost Analysis Project (EMSCAP). *Acad Emerg Med* 2012;19(2):210-216.

18. Petrie J, Easton S, Naik V, et al. Data from: Hospital costs of out-of-hospital cardiac arrest patients treated in intensive care; a single centre evaluation using the national tariff-based system. Dryad Digital Repository 2015. www.datadryad.org/resource/doi:10.5061/dryad.k78g1 (accessed 14 December 2018).

19. Benger J, Coates D, Davies S, et al. Randomised comparison of the effectiveness of the laryngeal mask airway supreme, i-gel and current practice in the initial airway management of out of hospital cardiac arrest: a feasibility study. *Br J Anaesth* 2016;116(2):262-8.

20. Nolan JP, Ferrando P, Soar J, et al. Increasing survival after admission to UK critical care units following cardiopulmonary resuscitation. *Crit Care* 2016;20(1):219.

21. Wan X, Wang W, Liu J, et al. Estimating the sample mean and standard deviation from the sample size, median, range and/or interquartile range. *BMC Med Res Methodol* 2014;14:135.

22. Phelps R, Dumas F, Maynard C, et al. Cerebral Performance Category and long-term prognosis following out-of-hospital cardiac arrest. *Crit Care Med* 2013;41(5):1252-7.

23. Office for National Statistics. National Life Tables 2015-2017. UK Statistics Authority 2018. www.ons.gov.uk/peoplepopulationandcommunity/birthsdeathsandmarriages/lifeexpectancies/datasets/nationallifetablesunitedkingdomreferencetables (accessed 14 December 2018).

For peer review only - http://bmjopen.bmj.com/site/about/guidelines.xhtml

24. Andrew E, Nehme Z, Wolfe R, et al. Long-term survival following out-of-hospital cardiac arrest. *Heart* 2017;103(14):1104-1110.

25. Department of Health. HCHS Pay and Price Series 2015/16. http://www.info.doh.gov.uk/doh/finman.nsf/af3d43e36a4c8f8500256722005b77f8/360a47827991d10a80258036002d8d9f?OpenDocument (accessed 14 December 2018).

26. Briggs A, Claxton K, Sculpher M. Decision modelling for health economic evaluation. Oxford: Oxford University Press 2006.

27. Department of Health, Reference Costs 2015-16. https://www.gov.uk/government/collections/nhs-reference-costs (accessed 14 December 2018).

28. Marti J, Hulme C, Ferreira Z, et al. The cost-effectiveness of a mechanical compression device in out-of-hospital cardiac arrest. *Resuscitation* 2017;117:1-7.

29. Moran PS, Teljeur C, Masterson S, et al. Cost-effectiveness of a national public access defibrillation programme. *Resuscitation* 2015;91:48-55.

30. Merchant RM, Becker LB, Abella BS, et al. Cost-effectiveness of therapeutic hypothermia after cardiac arrest. *Circ Cardiovasc Qual Outcomes* 2009;2(5):421-8.

31. Naess AC, Steen PA. Long term survival and costs per life year gained after out-of-hospital cardiac arrest. *Resuscitation* 2004;60(1):57-64.

32. Lai H, Choong CV, Fook-Chong S, et al. Interventional strategies associated with improvements in survival for out-of-hospital cardiac arrests in Singapore over 10 years. *Resuscitation* 2015;89:155-61.

33. Ginsberg GM, Kark JD, Einav S. Cost-utility analysis of treating out of hospital cardiac arrests in Jerusalem. *Resuscitation* 2015;86:54-61.

34. Sassi F. Calculating QALYs, comparing QALY and DALY calculations. *Health Policy Plan* 2006;21(5):402-8.

35. Pharoah PD, Sewell B, Fitzsimmons D, et al. Cost effectiveness of the NHS breast screening programme: life table model. *BMJ* 2013;346:f2618.

36. Hartwell D, Colquitt J, Loveman E, et al. Clinical effectiveness and cost-effectiveness of immediate angioplasty for acute myocardial infarction: systematic review and economic evaluation. *Health Technol Assess* 2005;9(17):1-99.

For peer review only - http://bmjopen.bmj.com/site/about/guidelines.xhtml

For peer review only

37. Nichol G, Brown S, Perkins G, et al. What change in outcomes after cardiac arrest is necessary to change practice? Results of an international survey. *Resuscitation* 2016;107:115-20.

38. Delgado MK, Staudenmayer K, Wang N, et al. Cost-Effectiveness of Helicopter Versus Ground Emergency Medical Services for Trauma Scene Transport in the United States. *Ann Emerg Med* 2013;62(4):351-364.

AUTHORS' CONTRIBUTIONS

JV designed the study protocol, collected and analysed the data and prepared the first draft of the manuscript.

JP reviewed the study protocol, supervised data analysis and reviewed and revised the manuscript.

JB supervised the research project and reviewed and revised the manuscript.

ACKNOWLEDGMENTS

We want to thank the South Western Ambulance Service NHS Foundation Trust and the Great Western Air Ambulance Charity for providing the data for this research. We are particularly grateful to the Patient and Public involvement group and the other participants in our previous research, whose views informed this research.

PREVIOUS PUBLICATIONS

This research was presented at the 999 Research Forum in Birmingham, United Kingdom, in April 2019.

DATA AVAILABILITY STATEMENT

The analytic model used for this research (TreeAge file) can be requested by sending an email to the author of this publication.

For peer review only - http://bmjopen.bmj.com/site/about/guidelines.xhtml

FIGURE LEGENDS

Figure 1. Final analytic model of prehospital critical care following out-of-hospital cardiac arrest, combining decision tree and Markov model. Due to limited space, the full pathway is shown for the Advanced Life Support option only.

Figure 2. Scatterplot of the cost-effectiveness of Advanced Life Support for out-of-hospital cardiac arrest, compared to no treatment.

QALY: Quality-adjusted life years

Figure 3. Cost-effectiveness acceptability curves of different plausible treatment effects of prehospital critical care for OHCA, when compared to ALS. The underlying baseline survival to hospital discharge for the ALS cohort in this model is 9.0%.

For peer review only

For peer review only - http://bmjopen.bmj.com/site/about/guidelines.xhtml

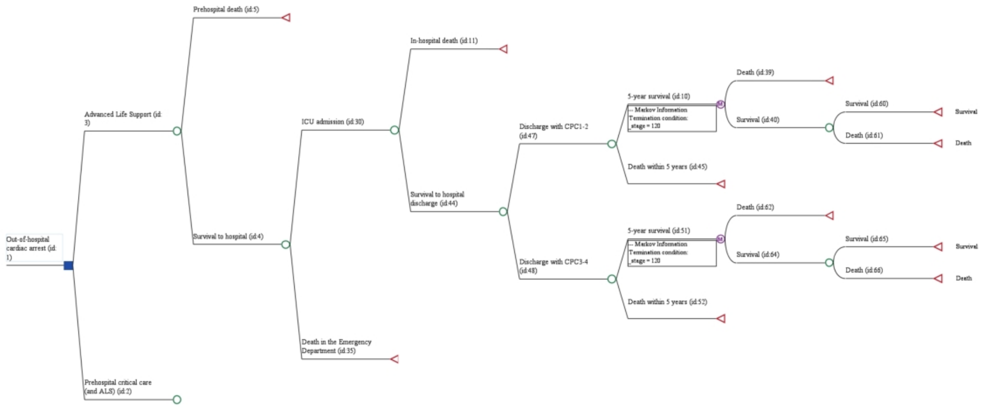

Figure 1. Final analytic model of prehospital critical care following out-of-hospital cardiac arrest, combining decision tree and Markov model. Due to limited space, the full pathway is shown for the Advanced Life Support option only.

255x110mm (300 x 300 DPI)

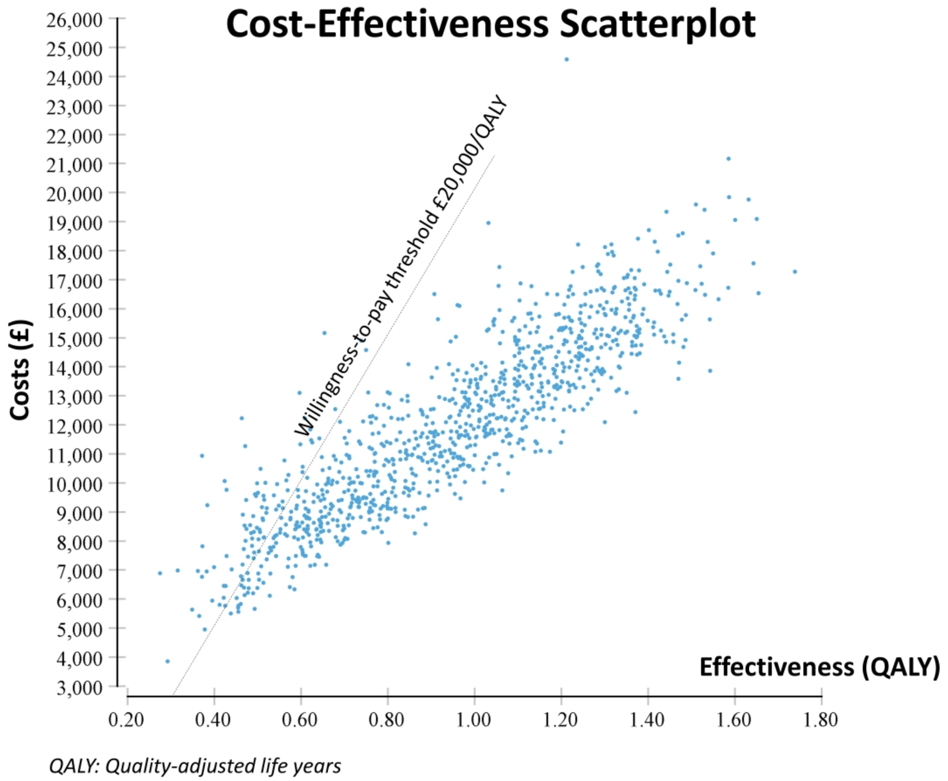

Figure 2. Scatterplot of the cost-effectiveness of Advanced Life Support for out-of-hospital cardiac arrest, compared to no treatment.

441x354mm (300 x 300 DPI)

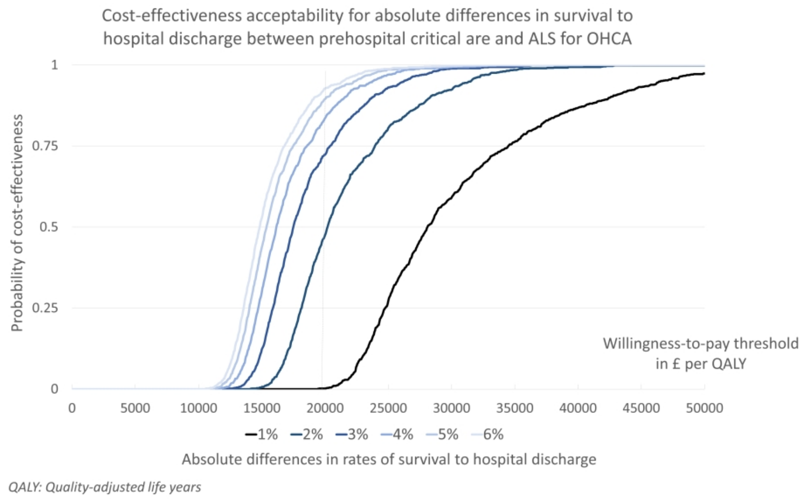

Figure 3. Cost-effectiveness acceptability curves of different plausible treatment effects of prehospital critical care for OHCA, when compared to ALS. The underlying baseline survival to hospital discharge for the ALS cohort in this model is 9.0%.

668x380mm (300 x 300 DPI)

Appendix 1. Search strategy and selection of publications for the synthesis of in-hospital costs of treatment for out-of-hospital cardiac arrest.

PubMed was searched using the search string *(((cost) OR economics) OR expenditure) AND "cardiac arrest"*, with search results limited to the last 10 years. Inclusion criteria were cost analysis of any intervention in adult, non-traumatic OHCA, in the UK. See Figure 1 for a flow chart of the search results.

Figure 1. Flow chart of the selection of publications. Search results from PubMed on 22 November 2017.

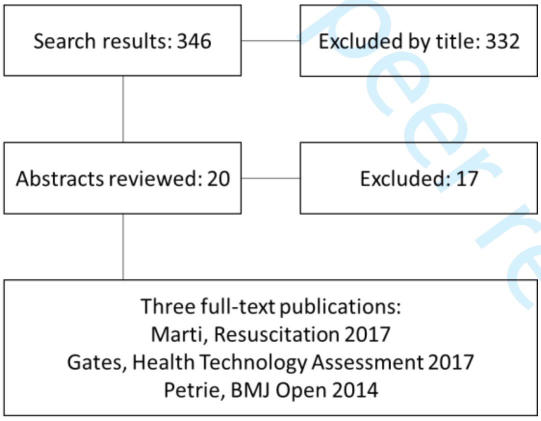

Twelve of the publications for which abstracts were reviewed were excluded as they were undertaken outside the UK, and were therefore expected to have significantly different cost and clinical parameters. The other five excluded studies did not include relevant costs. Of the three full-text publications reviewed, the studies by Marti et al. and Gates et al. related to the same randomised controlled trial of a mechanical chest compression device. Hospital treatment cost estimates were based on the length of stay of patients on the Intensive Care Unit (ICU) and on regular hospital wards as well as Emergency Department (ED) and outpatient costs. In contrast, Petrie et al. included additional significant costs of cardiac interventions, such as percutaneous coronary intervention (PCI), pacemaker insertion or coronary artery bypass graft surgery (CABG), resulting in an overall higher and probably more accurate estimate of hospital costs for OHCA. We therefore used the data from Petrie et al., including the study's open access source data, in combination with the Department of Health NHS Reference Costs 2015-16, for estimating the costs of in-hospital treatment. Post-discharge costs were extracted from Gates et al.

For peer review only - http://bmjopen.bmj.com/site/about/guidelines.xhtml

Appendix 2. Search strategy and selection of publications for the synthesis of quality and length of life after discharge from hospital following out-of-hospital cardiac arrest.

Quality and length of life after discharge from hospital following OHCA were estimated from previously published work. We undertook a focused literature search in PubMed, using the search string *(((quality of life[MeSH Terms]) OR utility) OR life expectancy[MeSH Terms]) AND cardiac arrest[MeSH Terms]*, with search results limited to the last 10 years. Inclusion criteria were adult non-traumatic OHCA and outcomes of either length or quality of life measured in utility. Where available, publications using data from the UK were included; where no UK data were available, data from North America or Australia were deemed sufficient. See Figure 1 for a flow chart of the literature review.

Figure 1. Focused literature search for data on length and quality of life following OHCA.

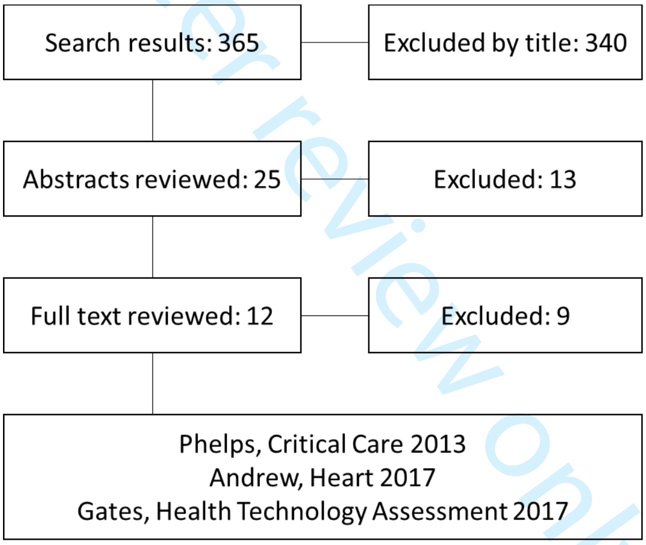

The two questions required for the decision tree and Markov model were

- What is the percentage of Cerebral Performance Category (CPC) 1-2 and CPC 3-4 amongst OHCA patients discharged from hospital?
- What is the quality and length of life of survivors of OHCA with CPC 1-2 and CPC 3-4, respectively?

Percentage of CPC 1-2 and CPC 3-4 at discharge from hospital

Gates et al. provided the most current and relevant information in their randomised controlled trial and embedded economic analysis of mechanical chest compressions during OHCA in the UK. CPC was

For peer review only

measured at three months after discharge from hospital, with 245 of 272 (90%) of patients achieving a CPC 1-2. This is somewhat higher than the 78% and 85% of CPC 1-2 survivors reported by Petrie et al. and Phelps et al. in a UK and Australian patient population, respectively. However, both these studies measured CPC at hospital discharge, rather than at three months after discharge. Patients discharged from hospital after OHCA with CPC 3-4 have a significantly higher mortality in the first months post discharge when compared to those discharged with CPC 1-2. Additional data from Phelps et al. at twelve months post discharge demonstrate that, of the 804 patients surviving to one year post discharge, 90% were discharged from hospital with CPC 1-2. We therefore concluded that 85% was a robust estimate for the percentage of OHCA survivors discharged with CPC 1-2.

Length of life after discharge from hospital

Phelps et al. also provide the most detailed description of long-term survival following OHCA, according to CPC at discharge from hospital, including person-years for the first year and the following four years after hospital discharge. We used these data to model mortality during the first 5 years after discharge from hospital following OHCA. Andrew et al. and Phelps et al. showed that survival rates after 5 years following discharge from hospital after OHCA were the same as those of the standard population. Mortality rates were therefore obtained from the National Life Tables for England and applied to the patient cohort from five years onwards. This resulted in an overall survival rate of 65.0% and 50.0% (CPC1-2) and 36.0% and 27.8% (CPC3-4) at 10 and 15 years post discharge, respectively.

Quality of life after discharge from hospital

In their RCT which recruited a total of 4,471 patients with OHCA between 2010 and 2013 in four UK ambulance services, Gates et al. reported health-related quality of life (HRQL) using the EuroQol – 5 dimension descriptive system (EQ-5D). The EQ-5D has been validated for measuring HRQL and for its use in economic evaluations in a variety of conditions, including cardiac arrest. Gates et al. converted their findings to health-state utilities using the UK tariff. Using a combination of decision tree and Markov model similar to that employed in this research, they calculated a utility 0.75 and 0.47 for the CPC 1-2 and CPC 3-4 groups, respectively. The use of EQ-5D for the calculation of utility is recommended by the National Institute for Health and Care Excellence.

For peer review only

CHEERS checklist—Items to include when reporting economic evaluations of health interventions

Section/item	Item No	Recommendation	Reported on page No/ line No
**Title and abstract**			
Title	1	Identify the study as an economic evaluation or use more specific terms such as "cost-effectiveness analysis", and describe the interventions compared.	Title, page 1
Abstract	2	Provide a structured summary of objectives, perspective, setting, methods (including study design and inputs), results (including base case and uncertainty analyses), and conclusions.	Page 2
**Introduction**			
Background and objectives	3	Provide an explicit statement of the broader context for the study.	Page 3-4
		Present the study question and its relevance for health policy or practice decisions.	Page 4
**Methods**			
Target population and subgroups	4	Describe characteristics of the base case population and subgroups analysed, including why they were chosen.	Page 5 'Clinical setting'
Setting and location	5	State relevant aspects of the system(s) in which the decision(s) need(s) to be made.	Page 5 'Clinical setting'
Study perspective	6	Describe the perspective of the study and relate this to the costs being evaluated.	Page 4 'Perspective and time horizon'
Comparators	7	Describe the interventions or strategies being compared and state why they were chosen.	Page 5 'Alternative interventions...'
Time horizon	8	State the time horizon(s) over which costs and consequences are being evaluated and say why appropriate.	Page 4 'Perspective and time horizon'
Discount rate	9	Report the choice of discount rate(s) used for costs and outcomes and say why appropriate.	Page 6 'Application of values...'
Choice of health outcomes	10	Describe what outcomes were used as the measure(s) of benefit in the evaluation and their relevance for the type of analysis performed.	Page 5-6 'Identification and synthesis...' Appendix 3
Measurement of effectiveness	11a	*Single study-based estimates:* Describe fully the design features of the single effectiveness study and why the single study was a sufficient source of clinical effectiveness data.	N/A
	11b	*Synthesis-based estimates*: Describe fully the methods used for identification of included studies and synthesis of clinical effectiveness data.	Page 5-6 'Identification and synthesis...' Appendices 2 to 3
Measurement and valuation of preference based outcomes	12	If applicable, describe the population and methods used to elicit preferences for outcomes.	Page 5-6 'Identification and synthesis...' Appendices 2 to 3
Estimating resources and costs	13a	*Single study-based economic evaluation:*Describe approaches used to estimate resource use associated with the alternative interventions. Describe primary or secondary research methods for valuing each resource item in terms of its unit cost. Describe any adjustments made to approximate to opportunity costs.	N/A

For peer review only - http://bmjopen.bmj.com/site/about/guidelines.xhtml

Section/item	Item No	Recommendation	Reported on page No/ line No
	13b	*Model-based economic evaluation:* Describe approaches and data sources used to estimate resource use associated with model health states. Describe primary or secondary research methods for valuing each resource item in terms of its unit cost. Describe any adjustments made to approximate to opportunity costs.	Page 5-6 'Identification and synthesis...' Appendices 2 to 3
Currency, price date, and conversion	14	Report the dates of the estimated resource quantities and unit costs. Describe methods for adjusting estimated unit costs to the year of reported costs if necessary. Describe methods for converting costs into a common currency base and the exchange rate.	Page 6 'Application of values...'
Choice of model	15	Describe and give reasons for the specific type of decision-analytical model used. Providing a figure to show model structure is strongly recommended.	Page 5 'Methods' Appendix 1
Assumptions	16	Describe all structural or other assumptions underpinning the decision-analytical model.	Page 5 'Methods' Appendices 1-3 Page 8 'Sensitivity analyses'
Analytical methods	17	Describe all analytical methods supporting the evaluation. This could include methods for dealing with skewed, missing, or censored data; extrapolation methods; methods for pooling data; approaches to validate or make adjustments (such as half cycle corrections) to a model; and methods for handling population heterogeneity and uncertainty.	Page 7, Table 1 Page 8 'Sensitivity analyses'
**Results**			
Study parameters	18	Report the values, ranges, references, and, if used, probability distributions for all parameters. Report reasons or sources for distributions used to represent uncertainty where appropriate. Providing a table to show the input values is strongly recommended.	Table 1
Incremental costs and outcomes	19	For each intervention, report mean values for the main categories of estimated costs and outcomes of interest, as well as mean differences between the comparator groups. If applicable, report incremental cost-effectiveness ratios.	Figure 2, Table , Table 4. We chose median rather than mean, due to the considerably skewed distributions
Characterising uncertainty	20a	*Single study-based economic evaluation* :Describe the effects of sampling uncertainty for the estimated incremental cost and incremental effectiveness parameters, together with the impact of methodological assumptions (such as discount rate, study perspective).	N/A
	20b	*Model-based economic evaluation:* Describe the effects on the results of uncertainty for all input parameters, and uncertainty related to the structure of the model and assumptions.	Page 7, Table 1
Characterising heterogeneity	21	If applicable, report differences in costs, outcomes, or cost-effectiveness that can be explained by variations between subgroups of patients with different baseline characteristics or other observed variability in effects that are not reducible by more information.	Figure 2, Table 3, Table 4
Study findings, limitations, generalisability, and	22	Summarise key study findings and describe how they support the conclusions reached. Discuss limitations	Page 12-14

For peer review only

Section/item	Item No	Recommendation	Reported on page No/ line No
current knowledge		and the generalisability of the findings and how the findings fit with current knowledge.	
**Other**			
Source of funding	23	Describe how the study was funded and the role of the funder in the identification, design, conduct, and reporting of the analysis. Describe other non-monetary sources of support.	Page 3
Conflicts of interest	24	Describe any potential for conflict of interest of study contributors in accordance with journal policy. In the absence of a journal policy, we recommend authors comply with International Committee of Medical Journal Editors recommendations.	Page 3

For consistency, the CHEERS statement checklist format is based on the format of the CONSORT statement checklist

For peer review only

BMJ Open

Cost-effectiveness of Advanced Life Support and prehospital critical care for out-of-hospital cardiac arrest in England: A decision analysis model

Journal:	*BMJ Open*
Manuscript ID	bmjopen-2018-028574.R2
Article Type:	Research
Date Submitted by the Author:	05-Jul-2019
Complete List of Authors:	von Vopelius-Feldt, Johannes; The University of the West of England, Faculty of Health and Applied Sciences; University Hospitals Bristol NHS Foundation Trust, Academic Department of Emergency Care<br>Powell, Jane; University of the West of England, Bristol, Centre for Public Health and Wellbeing<br>Benger, Jonathan; The University of the West of England, Faculty of Health and Applied Sciences; University Hospitals Bristol NHS Foundation Trust, Academic Department of Emergency Care
**Primary Subject Heading**:	Emergency medicine
Secondary Subject Heading:	Cardiovascular medicine, Health economics, Intensive care
Keywords:	ACCIDENT & EMERGENCY MEDICINE, Adult intensive & critical care < INTENSIVE & CRITICAL CARE, CARDIOLOGY

SCHOLARONE™
Manuscripts

Cost-effectiveness of Advanced Life Support and prehospital critical care for out-of-hospital cardiac arrest in England: A decision analysis model

AUTHORS

Johannes von Vopelius-Feldt[1,2] (ORCiD ID 0000-0002-6389-289X)

Jane Powell[3] (ORCiD ID 0000-0002-1828-1184)

Jonathan Benger[1,2] (ORCiD ID 0000-0001-6131-0916)

ASSOCIATIONS

1) Faculty of Health and Applied Sciences, University of the West of England, Bristol, United Kingdom

2) Academic Department of Emergency Care, University Hospitals Bristol NHS Foundation Trust, Bristol, United Kingdom

3) Centre for Public Health and Wellbeing, University of the West of England, Bristol, United Kingdom

CORRESPONDING AUTHOR

Johannes von Vopelius-Feldt

Emergency Care Research Group

1H14, Glenside Campus

Blackberry Hill, BS16 1DD Bristol

University of the West of England, Bristol

johannes.vonvopelius-feldt@uwe.ac.uk

Phone: 0044 (0)7958064069

WORD COUNT

3,141

For peer review only - http://bmjopen.bmj.com/site/about/guidelines.xhtml

ABSTRACT

Objectives

This research aimed to answer the following questions: What are the costs of prehospital Advanced Life Support (ALS) and prehospital critical care for out-of-hospital cardiac arrest (OHCA)? What is the cost-effectiveness of prehospital ALS? What improvement in survival rates from OHCA would prehospital critical care need to achieve in order to be cost-effective?

Setting

A single National Health Service (NHS) ambulance service and a charity funded prehospital critical care service in England.

Participants

The patient population is adult, non-traumatic OHCA.

Methods

We combined data from previously published research with data provided by a regional ambulance service and air ambulance charity to create a decision tree model, coupled with a Markov model, of costs and outcomes following OHCA. We compared no treatment for OHCA to the current standard of care of prehospital ALS, and prehospital ALS to prehospital critical care. To reflect the uncertainty in the underlying data, we used probabilistic and two-way sensitivity analyses.

Results

Costs of prehospital ALS and prehospital critical care were £347 and £1,711 per patient, respectively. When costs and outcomes of prehospital, in-hospital and post-discharge phase of OHCA care were combined, prehospital ALS was estimated to be cost-effective at £11,407 per quality-adjusted life year (QALY). In order to be cost-effective in addition to ALS, prehospital critical care for OHCA would need to achieve a minimally economically important difference (MEID) in survival to hospital discharge of 3-5%.

Conclusions

This is the first economic analysis to address the question of cost-effectiveness of prehospital critical care following OHCA. While costs of either prehospital ALS and/or critical care per patient with OHCA are relatively low, significant costs incur during hospital treatment and after discharge in patients who survive. Knowledge of the MEID for prehospital critical care can guide future research in this field.

Trial registration

ISRCTN registry ID ISRCTN18375201

For peer review only - http://bmjopen.bmj.com/site/about/guidelines.xhtml

STRENGTHS AND LIMITATIONS OF THIS STUDY

- This is the first study to describe the cost-effectiveness of the complete care pathway for out-of-hospital cardiac arrest in England
- This study uses a combination of primary and secondary data, therefore optimising the use of currently available evidence
- The decision analysis model includes a combination of probabilistic and two-way sensitivity analyses to address the complex question of cost-effectiveness of prehospital critical care in a transparent manner
- A limitation of this study is that is based on theoretic modelling only; the uncertainty in the underlying data is addressed by the methods and reflected in the presentation of results

FUNDING

This work is funded by a National Institute for Health Research (NIHR) doctoral research fellowship for Johannes von Vopelius-Feldt (DRF-2015-08-040). The funder is not involved in the design of the study or collection, analysis and interpretation of data, or in writing the manuscript. The views expressed are those of the author(s) and not necessarily those of the NHS, the NIHR or the Department of Health.

COMPETING INTEREST

JVVF and JB work as prehospital doctors with a regional prehospital critical care team.

INTRODUCTION

Rates of survival following out-of-hospital cardiac arrest (OHCA) remain low in the UK, with an overall survival rate to hospital discharge of 7.9%.[1] One area of potential improvement is the provision of prehospital care by the Emergency Medical Services (EMS).[2] The current standard of prehospital care in the UK is the provision of Advanced Life Support (ALS) by appropriately trained paramedics.[3] In an attempt to improve survival rates following OHCA, prehospital critical care teams (CCTs) are dispatched to OHCA in some regions in the UK, in addition to the standard ALS response.[4, 5] CCTs consist of specialist paramedics and/or doctors, with extended skills and competences, which we previously described in detail.[6] They are frequently based on air ambulances, with funding received variably through charitable donations and/or the National Health Service (NHS).[7]

For peer review only - http://bmjopen.bmj.com/site/about/guidelines.xhtml

For peer review only

The cost-effectiveness of ALS care provided by paramedics or physicians has been studied previously.[8, 9] However, generalisability of this evidence to current UK practice is limited due to the time elapse since the research was undertaken and considerable differences in EMS configurations. The cost-effectiveness of prehospital critical care for OHCA has not been researched in sufficient detail to guide funding decisions. A recently published systematic review of the effectiveness of CCTs for OHCA showed a potential small to moderate survival benefit but also identified issues limiting internal and external validity of the included observational studies.[10]

Economic analyses have been undertaken in regards to the cost-effectiveness of helicopter-based EMS care (HEMS) for trauma, with mixed results.[11, 12] To our knowledge, no economic analysis exists which addresses the question of cost-effectiveness of prehospital critical care for OHCA. In addition, a recent study of stakeholders' views of prehospital care for OHCA revealed contrasting opinion of whether current NHS funding of prehospital ALS care for OHCA represents an appropriate distribution of limited healthcare resources in the UK.[13] Adding to the complexity of cost-effectiveness of prehospital ALS or critical care for OHCA is the fact that the majority of costs occur along the patient care pathway in hospital or post-discharge.[14, 15]

In the absence of robust data regarding the effectiveness of prehospital ALS or critical care for OHCA in the UK setting, this study addresses the following questions

- What are the costs of prehospital ALS and prehospital care for OHCA in the UK?
- What is the cost-effectiveness of the current OHCA treatment pathway from prehospital ALS care to post-discharge costs, when compared to no treatment?
- What improvement in survival rates from OHCA would prehospital critical care need to achieve in order to be cost-effective, when compared to ALS?

METHODS

We combined data from previously published research with costs provided by a regional EMS provider and air ambulance charity to create a decision analysis model.

Development of the decision analysis model

The initial decision analysis model for this research considered three potential outcomes: prehospital death, in-hospital death or survival to hospital discharge. After a focused review of the literature, it became clear that differences in in-hospital treatment costs and survival with either poor or favourable neurological outcome have considerable impact on the cost-effectiveness estimates.[14, 15] We therefore expanded the model to include these important branches in a combination of

For peer review only - http://bmjopen.bmj.com/site/about/guidelines.xhtml

decision tree model for prehospital and hospital costs and outcomes, and a Markov model for post-discharge costs and outcomes. The structure is similar to the model used by Gates et al.[15] in their economic analysis of mechanical chest compression during prehospital care for OHCA. Figure 1 shows the final model (for simplicity, only the ALS arm of the model is displayed). Important assumptions, based on literature referenced in the sections below, are that

- A proportion of patients survive to hospital arrival but only receive palliative care, resulting in death in the emergency department or shortly afterwards.
- Patients who survive to hospital discharge with cerebral performance category (CPC) of 3-4 have a higher rate of survival following the first 5 years after hospital discharge, compared to those patients surviving with CPC 1-2.
- After 5 years post-discharge, annual survival rates for patients still alive at this point are the same as those of the average population.

Perspective and time horizon

In keeping with the recommendations of the National Institute for Health and Care Excellence (NICE), the perspective on costs chosen for this economic evaluation was that of the National Health Service (NHS) and personal social services.[16] The perspective on outcomes included all direct health effects on patients, namely length and quality of life (quality-adjusted life years, QALYs) with a corresponding long-term time horizon.

Clinical setting

The economic analysis was undertaken in the section of an NHS ambulance trust in England which was covered by a critical care team. It covers an area of 2.930 square miles with a population of approximately 2.5 million people. The area is largely suburban or rural with one metropolitan area and two urban areas. The overall population density is 860/square mile. The patient population is adult, non-traumatic OHCA.

Alternative interventions – Advanced life support and prehospital critical care

The NHS ambulance trust provided ALS paramedic care, using a combination of first responders, rapid response vehicles and ambulance. The section of the ambulance trust included in this analysis contained 27 ambulance stations with a total of approximately 800 ALS paramedics (employed part- or full-time) as well as emergency care assistants (ECAs) and ambulance technicians (data provided by ambulance trust). The median response time for OHCA during the study period was 7.4min (IQR 4.9min – 11.4min) and care on scene was provided by a mean of 2.43 core resources (paramedic-staffed double-crewed ambulances and/or rapid response vehicles). The prehospital critical care service was funded largely through an air ambulance charity but also received support from the

For peer review only - http://bmjopen.bmj.com/site/about/guidelines.xhtml

ambulance trust. It provided prehospital critical care cover from 7.00am to 1.00am, with access to a helicopter and rapid response vehicles. The team consisted of specialist paramedics in critical care and prehospital critical care doctors. The service responded to OHCAs in addition to the usual ambulance trust's ALS response.

Identification and synthesis of evidence

Costs

Financial data was obtained from the ambulance trust and air ambulance charity for the financial year 2015/16. This included pay for clinical and non-clinical staff as well as costs of dispatch infrastructure, ambulance stations, vehicles, fuel, medical supplies, communications equipment, community first responders and 3rd party costs. The amount of funding spent on OHCA was calculated as the number of OHCA cases divided by the number of cases of any aetiology. Calculating the proportion of resources spent on OHCA in this fashion distributes the costs of waiting time of resources in between emergency proportionally between OHCA cases and those of other aetiology.[17] The costs of in-hospital and post-discharge treatment were synthesised from relevant recent publications, identified by a focused systematic search of the literature (appendix 1).[14, 15, 18]

Outcomes

After a literature review (appendix 2), the following outcomes of interest were included in the decision analysis model (figure 1): survival to arrival at hospital; survival to ICU admission; survival to hospital discharge; CPC at discharge from hospital; 5-year survival following hospital discharge; annual mortality and quality of life. Prehospital outcomes after OHCA with ALS care were based on a one-year data sample provided by the participating ambulance trust (2016), while in-hospital outcomes were based on recent literature of OHCA in the UK.[19-21] For prehospital critical care, a range of effect sizes were simulated in two-way sensitivity analyses.[10] This approach was chosen to transparently reflect the considerable uncertainty of the effectiveness of prehospital critical care for OHCA in current research.[10] Post-discharge outcomes for the first five years after hospital discharge were retrieved from publications identified through a further focused systematic search of the literature (appendix 2).[15, 22] Further long-term outcomes (quality of life) were modelled according to survival rates provided by the UK's Office for National Statistics.[23, 24]

Application of values and distributions to the model

Prior to inclusion in the model, all costs were adjusted to Pound Sterling for the financial year 2016-17 according to the UK Hospital and Community Health Services (HCHS) pay and prices index.[25] In keeping with NICE guidelines, an annual discount rate of 3.5% was applied to costs and effects which occurred after the first year following discharge from hospital.[16] Table 1 provides a comprehensive

For peer review only - http://bmjopen.bmj.com/site/about/guidelines.xhtml

list of all model parameters, based on the sources of data described in the previous section. The confidence intervals or interquartile ranges of the data were transferred to sampling distributions.[26] In keeping with previous recommendations, we used Gamma distributions for costs and Beta distributions for probabilities.[26] Each parameter in table 1 corresponds to either a cost, effect or probability of each chance node or Markov node of the decision analysis model in figure 1. For costs and effects, these are simply added up for each potential outcome. For example, in-hospital death after prehospital critical care would incur the costs of prehospital critical care plus ALS, costs of ED treatment, and ICU and non-ICU costs of non-survivors. The effect is 0 QALYs, as the outcome is death. The probabilities for each node were entered into the model as the values described in table 1, with the exception of the probability of survival to hospital discharge. To create a 10% rate of survival to hospital discharge after prehospital critical care, for example, the probability value for the corresponding node would need to be entered as a function of '10%, dividided by *p ICU admission*, divided by *p survival to hospital'*.

For peer review only

For peer review only - http://bmjopen.bmj.com/site/about/guidelines.xhtml

Table 1. Values and distributions of parameters of the decision tree and Markov model; all costs adjusted for inflation to the financial year 2016-17

Parameters	Cohort / patient group	Mean value (95% confidence interval)	Distribution	Source
**Prehospital (decision tree model)**				
Costs of prehospital care	ALS	£347	Gamma	Own data
	Critical care (in addition to ALS)	£2,058 (£1,711 critical care plus £347 ALS care)	N/A	
p survival to hospital	ALS	0.26 (0.25 – 0.27)	Beta	von Vopelius-Feldt [10]
	Critical care	0.25 – 0.31	N/A	
**In-hospital (decision tree model)**				
Costs of ED treatment		£377 (£355 – £399)	Gamma	Department of Health [27]
p ICU admission*		0.65 (0.58 – 0.71)	Beta	Benger [19]
Costs of ICU treatment (daily) and length of stay on ICU (LOS)	Survival to hospital discharge	£1,745 (£1,654 – £1,836)<br>LOS (days) 5.7 (5.4 – 6.0)	Gamma	Petrie [14]<br>Nolan [20]
	In-hospital death	£1,768 (£1,668 – £1,868)<br>LOS (days) 2.4 (2.3 – 2.5)		
Cost of non-ICU treatment (total)	Survival to hospital discharge	£12,823 (£10,123 – £15,523)	Gamma	Petrie [14]<br>Nolan [20]
	In-hospital death	£3,835 (£3,045 – £4,625)		
p survival to hospital discharge**	ALS	0.09 (0.08 – 0.10)	Beta	von Vopelius-Feldt [10]
	Critical care	0.10 – 0.15	N/A	
**Post-discharge (decision tree model)**				
p survival with CPC1-2***		0.85 (0.826 – 0.871)	Beta	Phelps [22]
p 5-year survival	CPC1-2	0.740 (0.709 – 0.768)	Beta	Phelps [22]
	CPC3-4	0.408 (0.332 – 0.489)		
Person-years per death within 5 years	CPC1-2	1.827 (1.750 – 1.878)	Gamma	Phelps [22]
	CPC3-4	0.313 (0.254 – 0.375)		
Utility	CPC1-2	0.75 (0.70 – 0.80)	Beta	Gates [15]
	CPC3-4	0.47 (0.42 – 0.52)		
Annual healthcare costs	CPC1-2	£3,358	Gamma	Gates [15]
	CPC3-4	£43,670		
**Long-term outcomes (Markov model)**				
p annual mortality after 5 years post discharge	CPC1-2<br>CPC3-4	National age-adjusted mortality rates	Exact values	Office for National Statistics [23], Andrew [24]

*of survivors to hospital **of all cases of OHCA ***of survivors to hospital discharge

ALS: Advanced Life Support, N/A: not applicable, p: probability, ED: emergency department, ICU: intensive care unit, LOS: length of stay, CPC: Cerebral Performance Category

For peer review only

For peer review only - http://bmjopen.bmj.com/site/about/guidelines.xhtml

Sensitivity analyses

As the analysis was based largely on secondary data, we undertook a probabilistic sensitivity analysis of the decision analysis model, to reflect the underlying uncertainty in the data.[26] We ran 1,000 iterations of the decision analysis model in a Monte Carlo simulation. For each iteration, the values of all model parameters were drawn at random from the sampling distributions described in table 1. In addition, the probabilities of survival to hospital admission and survival to hospital discharge after prehospital critical care were subject to a two-way sensitivity analysis, reflecting a range of potential effects based on a recent systematic review.[10] All analysis was undertaken in TreeAge (Version 2017).

Model validation

Validity of decision analysis models can be examined in a number of different ways.[26] Face validity was examined by presenting the model, values and results to a group of prehospital clinicians and air ambulance charity staff. Internal validity was assessed through the sensitivity analyses described above, as well as selecting extreme values for each individual parameter in the model and checking if the results were influenced as expected. We also undertook visual inspection of the values selected by the software for each variable during the probabilistic sensitivity, to assure that values were in the ranges expected for the parameter. While the software produced an error message if probabilities either did not add up to 1 or had individual values of less than 0 or more than 1, we also hand checked all probabilities to assure that the overall probability of death / survival was correctly calculated from the probabilities at each step of the decision tree model.

Presentation of results

In keeping with the three research questions outlined in the introduction, the results will be presented in three sections. First, we will describe the costs occurring during prehospital care for OHCA, for both ALS and critical care. Outcome measure will be Pound Sterling (£). We will then describe the cost-effectiveness of prehospital ALS, including the complete patient pathway, when compared to no treatment. The outcome measure will be the incremental cost-effectiveness in £/QALY. Finally, we will analyse the MEID which prehospital critical care would need to achieve in order to be cost effective at a willingness-to-pay (WTP) threshold of £20,000/QALY. The outcome measure will be the absolute difference in survival to hospital discharge between ALS and prehospital critical care which fulfils the MEID criteria.

Patient and public involvement

A Patient and Public Involvement (PPI) Group was consulted during the application and planning process of the doctoral research fellowship which underlies this research. In addition, the PPI group's

For peer review only - http://bmjopen.bmj.com/site/about/guidelines.xhtml

as well as other relevant stakeholders' views expressed during previous research[13] informed the methods of this research. Results will be disseminated to all stakeholders who participated, through an executive summary of this research.

Ethics and consent

With the exception of the financial data of prehospital care costs, all data were obtained from previously published research or government documents. Therefore, patient consent was not required. The research was reviewed and approved by the Sheffield National Research Ethics Committee, York and Humber on 29 July 2016, reference number 16/YH/0300.

RESULTS

Prehospital costs

Table 2 gives an overview of the costs of prehospital ALS care and prehospital critical care for OHCA. The total expenditure for ALS care is over 25 times higher than the costs of providing prehospital critical care for the same geographical area. However, OHCAs represent approximately one third of the work load of the prehospital critical care team, whereas only 0.85% of ALS care funding can be attributed to OHCA care. The average cost of a patient with OHCA being attended by ALS paramedics is £347, while the cost for attendance of a prehospital critical care team is £1,711 (plus £347, as ALS resources would still be dispatched with critical care team attendance).

For peer review only - http://bmjopen.bmj.com/site/about/guidelines.xhtml

For peer review only

Table 2. Prehospital treatment costs for out-of-hospital cardiac arrest

Total expenditure 2015-16 (in £)		
	**ALS care**	**Critical care**
Staff costs	36,732,844	665,293
Vehicles (including fuel)	8,145,245	13,659
Helicopter (including fuel)	-	1,318,432
Buildings	2,705,442	37,693
Equipment	1,681,383	63,296
Other	6,261,901	37,752
Dispatch centre	2,226,955	45,000
**Total**	**57,753,770**	**2,181,125**
**Expenditure per OHCA 2015-16 (in £)**		
**Percentage of resources devoted to OHCA**	0.85%	30%
**Number of OHCA patients**	1412	388
**Cost per OHCA patient**	**347**	**1,711**

Cost-effectiveness of Advanced Life Support for OHCA

The estimated cost-effectiveness of the current standard pathway for OHCA, including prehospital ALS, hospital admission, ICU and non-ICU treatment, as well as post-discharge healthcare costs, is £11,407/QALY (median, IQR £6,840 /QALY – £16,863/QALY), when compared to no treatment. Figure 2 shows the corresponding scatterplot with results from 1,000 iterations of the probabilistic sensitivity analysis. As the majority of these 1,000 results lie below the WTP threshold of £20,000/QALY (approximately 93%), prehospital ALS for OHCA can be assumed to be cost-effective at this WTP threshold with a high degree of certainty.

Incremental cost-effectiveness of prehospital critical care for OHCA

Figure 3 shows the cost-effectiveness acceptability curves for a number of potential treatment effects of prehospital critical care following OHCA. For this model, the ratio of rates of survival to arrival at hospital and survival to hospital discharge in the prehospital critical care treatment arm was kept constant. Rates of survival to hospital arrival and of survival to hospital discharge after ALS care for OHCA were 25.8% and 9.0%, respectively. The intersections of the WTP threshold and the cost-acceptability curves provide the probability of prehospital critical care being cost-effective. Prehospital critical care needs to achieve a 2% or 3% absolute increase in survival to hospital discharge to have a 48% or 72% probability of being cost effective at a WTP threshold of £20,000 per QALY, respectively. With a lower absolute difference in survival rates of 1%, prehospital critical care has a less than 1% probability of being cost effective at the same WTP threshold. Higher absolute differences

For peer review only - http://bmjopen.bmj.com/site/about/guidelines.xhtml

in survival of 4%, 5% and 6% result in probabilities of cost-effectiveness at the WTP threshold of £20,000 of 84%, 90% and 93%, respectively.

The results of the analysis presented in figure 3 assume that the rates of survival to hospital increase proportionally with the rates of survival to hospital discharge. However, as the majority of costs for the care of OHCA is generated by in-hospital treatments, variations in the rate of survival to hospital can have considerable influence on costs, independently of later survival to hospital discharge. Table 3 provides estimates and interquartile ranges for possible combinations of survival to hospital and survival to hospital discharge rates. As table 3 demonstrates, higher rates of survival to hospital arrival result in moderate increases in incremental costs of prehospital critical care for OHCA.

Table 3. Incremental cost-effectiveness of prehospital critical care for OHCA (in £ per quality-adjusted life year), for different rates of survival to hospital and survival to hospital discharge, compared to ALS.

**Difference in rates of survival to hospital**	**Difference in rates of survival to hospital discharge (median, 95% CI)**					
	**+1%**	**+2%**	**+3%**	**+4%**	**+5%**	**+6%**
**-5%**	25,000 (16,500 – 46,000)	18,000 (12,500 – 28,500)	15,500 (11,000 – 23,500)	14,500 (10,500 – 21,000)	Not achievable	Not achievable
**0%**	**27,500 (20,000 – 47,500)**	19,000 (14,500 – 30,000)	16,500 (12,500 – 24,500)	15,000 (11,500 – 22,000)	14,000 (11,000 – 20,000)	13,500 (10,500 – 19,000)
**+5%**	30,500 (21,500 – 53,000)	**20,500 (15,500 – 33,500)**	17,000 (13,000 – 26,500)	15,500 (12,000 – 23,000)	14,500 (11,500 – 21,500)	14,000 (11,000 – 20,000)
**+10%**	33,500 (22,500 – 61,000)	22,000 (16,000 – 37,500)	**18,000 (14,000 – 28,500)**	**16,500 (12,500 – 24,500)**	15,000 (12,000 – 22,000)	14,500 (11,500 – 21,000)
**+15%**	36,500 (23,000 – 70,500)	23,500 (17,000 – 42,000)	20,000 (14,500 – 32,000)	17,000 (13,000 – 26,500)	**16,000 (12,500 – 23,500)**	**15,000 (11,500 – 22,000)**
**+20%**	39,500 (23,500 – 79,500)	24,500 (17,000 – 46,000)	20,000 (15,000 – 34,000)	17,500 (13,500 – 29,000)	16,500 (12,500 – 25,500)	15,500 (12,000 – 23,000)

Light and darker shades of grey indicate most likely combination of differences in rates of survival to hospital and survival to hospital discharge. All costs are in £ and have been rounded to the nearest £500 value.

Table 4 presents a further sensitivity analysis of the effects of varying costs of prehospital critical care and rates of survival to hospital discharge. Variations in the costs of prehospital critical care for OHCA only have a minor to moderate effect on its cost-effectiveness.

For peer review only - http://bmjopen.bmj.com/site/about/guidelines.xhtml

Table 4. Incremental cost-effectiveness of prehospital critical care for OHCA (in £ per quality-adjusted life year), for a range of costs of prehospital critical care and different rates of survival to hospital discharge, compared to ALS.

**Difference in costs of prehospital care**	**Difference in rates of survival to hospital discharge (median, 95% CI)**					
	**+1%**	**+2%**	**+3%**	**+4%**	**+5%**	**+6%**
**-50%**	20,000 (16,000 – 33,500)	16,000 (13,000 – 25,500)	15,000 (12,000 – 23,000)	14,000 (11,500 – 21,500)	14,000 (11,000 – 21,000)	13,500 (11,000 – 20,500)
**-20%**	25,000 (19,000 – 43,000)	18,500 (14,500 – 30,500)	16,500 (13,000 – 26,000)	15,500 (12,500 – 24,000)	15,000 (12,000 – 23,000)	14,500 (11,500 – 22,000)
**0%**	**28,000 (21,500 – 50,500)**	**20,000 (16,000 – 33,500)**	**17,500 (14,000 – 28,500)**	**16,000 (13,000 – 25,500)**	**15,500 (12,500 – 24,000)**	**15,000 (12,000 – 23,000)**
**+20%**	31,500 (24,000 – 57,500)	22,000 (17,000 – 37,000)	18,500 (14,500 – 30,500)	17,000 (13,500 – 27,000)	16,000 (13,000 – 25,500)	15,500 (12,500 – 24,000)
**+50%**	36,000 (27,500 – 67,500)	24,00 (18,500 – 41,500)	20,000 (16,000 – 33,500)	18,000 (14,500 – 30,000)	17,000 (13,500 – 27,000)	16,000 (13,000 – 25,500)

All costs are in £ and have been rounded to the nearest £500 value.

DISCUSSION

In this decision model analysis, the cost-effectiveness of prehospital ALS care or prehospital critical care for OHCA was determined to a considerable degree by their effects on short and long-term survival, with the majority of costs incurring during hospital treatment or post-discharge.

Prehospital costs vs later costs

Given the complexity of the economic analysis of the whole OHCA pathway, it is worth first looking at prehospital resource use only. The ambulance service described in this analysis had an annual spending of over 50 million pounds sterling. However, during the study period, patients with OHCA only represented 0.22% of all emergency calls attended. Even with the requirement for more resources per OHCA patient compared to most other prehospital conditions, only 0.82% of the annual spending was allocated to OHCAs, at an estimated cost of £347 per OHCA. This might seem a rather low amount of money to be spent on the immediate care for a life or death situation.[13]

For peer review only - http://bmjopen.bmj.com/site/about/guidelines.xhtml

In contrast, the annual costs of providing a helicopter-based prehospital critical care team, covering the same geographic area was relatively low, at just over 2 million pounds per year. Due to the fact that the critical care team attended much fewer patients (approximately 1,200 during the study period) with a higher proportion of OHCAs (30%), the incremental costs of prehospital critical care per OHCA were estimated at £1,711. Given the gravity of the acute situation of an OHCA, this incremental cost might be considered an effective use of EMS resources.

Focusing only on prehospital costs of OHCA would make comparison to other healthcare interventions difficult, and would ignore the importance of costs accumulating further along the OHCA pathway. For patients with OHCA, where resuscitation is unsuccessful and who are declared dead on scene, no further costs occur. However, a large proportion (25.8% in this cohort) of patients with OHCA survive the prehospital phase of their care and are admitted to a hospital. We estimated the costs of in-hospital treatment to be approximately £22,000 for patients surviving to hospital discharge and £8,500 for patients who die in hospital.[14, 20] The major contributors to these costs are ICU-bed days (all patients) and interventions such as PPCI, ICD implantation or coronary artery bypass graft surgery (almost exclusively in survivors).[14] Further costs can accumulate after hospital discharge, most importantly the long-term care services required for the small proportion of patients who survive to hospital discharge with poor neurological function (over £40,000 per year).[15]

Cost-effectiveness of Advanced Life Support for OHCA

We estimated the cost-effectiveness of paramedic-delivered ALS for OHCA to be approximately £11,500 per QALY. With the upper limit of the interquartile range at approximately £16,800, this makes ALS for OHCA almost certainly cost-effective at a NICE WTP threshold of £20,000 per QALY.[16]

While a number of previous publications address the cost-effectiveness of individual aspects of prehospital care or hospital care for OHCA,[28-30] only few address the cost-effectiveness of ALS for OHCA in the context of the complete patient pathway. Naess et al.[31] estimated the cost-effectiveness of ALS for OHCA in Norway to be approximately £6,000 per QALY in 2004. This much lower estimate is likely due to inflation since the year of publication but also development of in-hospital cardiac arrest care with higher rates of interventions in modern OHCA care, and the costs associated with this.[20, 32] More recently, Ginsberg et al.[33] reported the cost-effectiveness of ALS for OHCA to be $28,864 per Disability Adjusted Life Year (DALY) averted, in an Israeli EMS, corresponding to approximately £15,000 - £28,500 per QALY.[34] Despite some uncertainty of the data underlying this analysis, and taking previous research findings into account, we can be fairly certain that paramedic-delivered ALS is a cost-effective treatment for OHCA in the UK. In addition, In

For peer review only - http://bmjopen.bmj.com/site/about/guidelines.xhtml

terms of £ per QALY, it compares favourably with a range of interventions currently funded by the NHS.[35, 36]

Costs of prehospital critical care

As the current observational research describing the effectiveness of prehospital critical care for OHCA is inconsistent and likely suffers from confounding,[10] we decided not to include it in this probabilistic sensitivity analysis. Instead, we used a range of potential absolute effect sizes on rates of survival to hospital discharge, ranging from 1% to 6%, reflecting the most likely range of effect sizes.[10] This sensitivity analysis also allowed us to determine a minimally economically important difference (MEID) in survival rates which would need to be achieved by prehospital critical care services in order to be cost-effective.

At a WTP threshold of £20,000 per QALY, the MEID is in the range of 3-5% absolute improvement in survival to hospital discharge after OHCA. Any improvement in survival rates after OHCA of less than 3%, caused by prehospital critical care services, is very unlikely to be considered cost-effective within an NHS setting.[16] The exact value for the MEID depends on how certain stakeholders want to be about the cost-effectiveness estimate at a given WTP (figure 3), the rates of survival to hospital admission (table 3) and the exact costs of a given prehospital critical care service (table 4). The MEID can be used to guide sample size calculations of future research and to frame the expectations or aims of stakeholders and prehospital critical care services providing care for OHCA.[13] Interestingly, the MEID of prehospital critical care for OHCA calculated in this analysis equals the minimally clinically important difference (MCID) in survival following OHCA described in previous research.[37]

LIMITATIONS

This economic analysis is based on a theoretical construct of the costs and effects of the care pathway for OHCA. As such, assumptions about events occurring in reality had to be made, and have to be assumed to reflect reality accurately, in order for the model to be internally valid.[26] On the other hand, using a modelling approach allowed us to incorporate a considerable number of data sources and to undertake a range of sensitivity analyses, which improves generalisability. While we undertook steps to assure face validity and internal validity of the model, we are unable to reliable test external validity (through simulation of real life events).

We did not adapt a micro-costing approach which would have, for example, included the exact number of paramedics at scene for an OHCA or the amount of drugs or equipment used. Such an approach would have likely resulted in an overall more accurate estimate of the costs of ALS care for

For peer review only - http://bmjopen.bmj.com/site/about/guidelines.xhtml

OHCA, but would have required tremendous efforts to collect and analyse the data. As costs occurring in the later stages of care for OHCA were much higher than during the prehospital phase, a micro-costing approach would have been unlikely to change the cost estimate for prehospital care significantly. Reassuringly, the estimated cost of £347 for ALS care for OHCA compares realistically to the NHS National Schedule of Reference Costs of an average of £236 for *'see and treat and convey'* ambulance activities (considering the higher resource use for OHCA patients).[27]

The main driver of costs for prehospital critical care was the helicopter, followed by staffing costs. We are aware of a number of prehospital critical care services which attend OHCAs using only ground vehicles. This would certainly reduce overall costs of the service (and is therefore reflected in the sensitivity analysis) but also the number of OHCAs which the CCT could reach, particular in rural/suburban areas. In regards to the validity of the overall model, there are few previous publications to compare to. Reassuringly, Delgado et al.[38] used a similar decision model analysis approach to examine the potential cost-effectiveness of HEMS for trauma cases in the US. They found that a 3.7% absolute decrease in mortality was required for HEMS to be cost-effective at a WTP of $50,000 (£38,000) per QALY.

This analysis focuses exclusively on adult, non-traumatic OHCA, as OHCA due to trauma or occurring in the paediatric population is much rarer and due to distinctively different pathology. Results should not be extrapolated to these situations.

CONCLUSION

This is the first economic analysis, based on a decision analysis model, to address the question of cost-effectiveness of prehospital critical care following OHCA. While costs of either prehospital ALS and/or critical care per patient with OHCA are relatively low, significant costs incur during hospital treatment and after discharge in patients who survive. Taking the whole OHCA care pathway into account, prehospital ALS as currently delivered in most NHS ambulance trusts is very likely to be cost-effective at a WTP of £20,000 per QALY. For prehospital critical care to be cost-effective at the same WTP threshold, it would need to improve survival from OHCA by approximately 3-5%.

For peer review only - http://bmjopen.bmj.com/site/about/guidelines.xhtml

REFERENCES

1. Hawkes C, Booth S, Ji C, et al. Epidemiology and outcomes from out-of-hospital cardiac arrests in England. *Resuscitation* 2017;110:133-140.

2. Boyce LW, Vliet Vlieland TP, Bosch J, et al. High survival rate of 43% in out-of-hospital cardiac arrest patients in an optimised chain of survival. *Neth Heart J* 2015;23(1):20-5.

3. Brown S, Kumar D, Millins M, et al. UK ambulance services clinical practice guidelines 2016. Bridgewater: Class Professional 2016.

4. von Vopelius-Feldt J, Benger J. Critical care paramedics in England: a national survey of ambulance services. *Eur J Emerg Med* 2014;21(4):301-4.

5. Hyde P, Mackenzie R, Ng G, et al. Availability and utilisation of physician-based pre-hospital critical care support to the NHS ambulance service in England, Wales and Northern Ireland. *Emerg Med J*, 2012;29(3):177-181.

6. von Vopelius-Feldt, J. and J. Benger, Who does what in prehospital critical care? An analysis of competencies of paramedics, critical care paramedics and prehospital physicians. *Emerg Med J* 2014;31(12):1009-13.

7. Association of Air Ambulances Annual Report 2016. Henley in Arden: Association of Air Ambulances 2017.

8. Nichol G, Laupacis A, Stiell IG, et al. Cost-effectiveness analysis of potential improvements to emergency medical services for victims of out-of-hospital cardiac arrest. *Ann Emerg Med* 1996;27(6):711-20.

9. Yen ZS, Chen YT, Ko PC, et al. Cost-effectiveness of different advanced life support providers for victims of out-of-hospital cardiac arrests. *J Formos Med Assoc* 2006;105(12):1001-7.

10. von Vopelius-Feldt J, Brandling J, Benger J. Systematic review of the effectiveness of prehospital critical care following out-of-hospital cardiac arrest. *Resuscitation* 2017;114:40-46.

11. Taylor CB, Stevenson M, Jan S, et al. A systematic review of the costs and benefits of helicopter emergency medical services. *Injury* 2010;41(1):10-20.

12. Snooks HA, Nicholl JP, Brazier JE, et al. The costs and benefits of helicopter emergency ambulance services in England and Wales. *J Public Health Med* 1996;18(1):67-77.

For peer review only

For peer review only - http://bmjopen.bmj.com/site/about/guidelines.xhtml

13. Von Vopelius-Feldt J, Brandling J, Benger J. Variations in stakeholders' priorities and views onrandomisation and funding decisions in out-of-hospital cardiacarrest: An exploratory study. *Health Sci Rep* 2018;1(9):e78.

14. Petrie J, Easton S, Naik V, et al. Hospital costs of out-of-hospital cardiac arrest patients treated in intensive care; a single centre evaluation using the national tariff-based system. *BMJ Open* 2015;5(4):e005797.

15. Gates S, Lall R, Quinn T, et al. Prehospital randomised assessment of a mechanical compression device in out-of-hospital cardiac arrest (PARAMEDIC): a pragmatic, cluster randomised trial and economic evaluation. *Health Technol Assess* 2017;21(11):1-176.

16. National Institute for Health and Care Excellence. Guide to the methods of technology appraisal 2013. www.nice.org.uk/process/pmg9 (accessed 14 December 2018).

17. Lerner EB, Garrison HG, Nichol G, et al. An Economic Toolkit for Identifying the Cost of Emergency Medical Services (EMS) Systems: Detailed Methodology of the EMS Cost Analysis Project (EMSCAP). *Acad Emerg Med* 2012;19(2):210-216.

18. Petrie J, Easton S, Naik V, et al. Data from: Hospital costs of out-of-hospital cardiac arrest patients treated in intensive care; a single centre evaluation using the national tariff-based system. Dryad Digital Repository 2015. www.datadryad.org/resource/doi:10.5061/dryad.k78g1 (accessed 14 December 2018).

19. Benger J, Coates D, Davies S, et al. Randomised comparison of the effectiveness of the laryngeal mask airway supreme, i-gel and current practice in the initial airway management of out of hospital cardiac arrest: a feasibility study. *Br J Anaesth* 2016;116(2):262-8.

20. Nolan JP, Ferrando P, Soar J, et al. Increasing survival after admission to UK critical care units following cardiopulmonary resuscitation. *Crit Care* 2016;20(1):219.

21. Wan X, Wang W, Liu J, et al. Estimating the sample mean and standard deviation from the sample size, median, range and/or interquartile range. *BMC Med Res Methodol* 2014;14:135.

22. Phelps R, Dumas F, Maynard C, et al. Cerebral Performance Category and long-term prognosis following out-of-hospital cardiac arrest. *Crit Care Med* 2013;41(5):1252-7.

23. Office for National Statistics. National Life Tables 2015-2017. UK Statistics Authority 2018. www.ons.gov.uk/peoplepopulationandcommunity/birthsdeathsandmarriages/lifeexpectancies/datasets/nationallifetablesunitedkingdomreferencetables (accessed 14 December 2018).

For peer review only - http://bmjopen.bmj.com/site/about/guidelines.xhtml

24. Andrew E, Nehme Z, Wolfe R, et al. Long-term survival following out-of-hospital cardiac arrest. *Heart* 2017;103(14):1104-1110.

25. Department of Health. HCHS Pay and Price Series 2015/16. http://www.info.doh.gov.uk/doh/finman.nsf/af3d43e36a4c8f8500256722005b77f8/360a47827991d10a80258036002d8d9f?OpenDocument (accessed 14 December 2018).

26. Briggs A, Claxton K, Sculpher M. Decision modelling for health economic evaluation. Oxford: Oxford University Press 2006.

27. Department of Health, Reference Costs 2015-16. https://www.gov.uk/government/collections/nhs-reference-costs (accessed 14 December 2018).

28. Marti J, Hulme C, Ferreira Z, et al. The cost-effectiveness of a mechanical compression device in out-of-hospital cardiac arrest. *Resuscitation* 2017;117:1-7.

29. Moran PS, Teljeur C, Masterson S, et al. Cost-effectiveness of a national public access defibrillation programme. *Resuscitation* 2015;91:48-55.

30. Merchant RM, Becker LB, Abella BS, et al. Cost-effectiveness of therapeutic hypothermia after cardiac arrest. *Circ Cardiovasc Qual Outcomes* 2009;2(5):421-8.

31. Naess AC, Steen PA. Long term survival and costs per life year gained after out-of-hospital cardiac arrest. *Resuscitation* 2004;60(1):57-64.

32. Lai H, Choong CV, Fook-Chong S, et al. Interventional strategies associated with improvements in survival for out-of-hospital cardiac arrests in Singapore over 10 years. *Resuscitation* 2015;89:155-61.

33. Ginsberg GM, Kark JD, Einav S. Cost-utility analysis of treating out of hospital cardiac arrests in Jerusalem. *Resuscitation* 2015;86:54-61.

34. Sassi F. Calculating QALYs, comparing QALY and DALY calculations. *Health Policy Plan* 2006;21(5):402-8.

35. Pharoah PD, Sewell B, Fitzsimmons D, et al. Cost effectiveness of the NHS breast screening programme: life table model. *BMJ* 2013;346:f2618.

36. Hartwell D, Colquitt J, Loveman E, et al. Clinical effectiveness and cost-effectiveness of immediate angioplasty for acute myocardial infarction: systematic review and economic evaluation. *Health Technol Assess* 2005;9(17):1-99.

For peer review only

For peer review only - http://bmjopen.bmj.com/site/about/guidelines.xhtml

37. Nichol G, Brown S, Perkins G, et al. What change in outcomes after cardiac arrest is necessary to change practice? Results of an international survey. *Resuscitation* 2016;107:115-20.

38. Delgado MK, Staudenmayer K, Wang N, et al. Cost-Effectiveness of Helicopter Versus Ground Emergency Medical Services for Trauma Scene Transport in the United States. *Ann Emerg Med* 2013;62(4):351-364.

AUTHORS' CONTRIBUTIONS

JV designed the study protocol, collected and analysed the data and prepared the first draft of the manuscript.

JP reviewed the study protocol, supervised data analysis and reviewed and revised the manuscript.

JB supervised the research project and reviewed and revised the manuscript.

ACKNOWLEDGMENTS

We want to thank the South Western Ambulance Service NHS Foundation Trust and the Great Western Air Ambulance Charity for providing the data for this research. We are particularly grateful to the Patient and Public involvement group and the other participants in our previous research, whose views informed this research.

PREVIOUS PUBLICATIONS

This research was presented at the 999 Research Forum in Birmingham, United Kingdom, in April 2019.

DATA AVAILABILITY STATEMENT

The analytic model used for this research (TreeAge file) can be requested by sending an email to the author of this publication.

For peer review only

FIGURE LEGENDS

Figure 1. Final analytic model of prehospital critical care following out-of-hospital cardiac arrest, combining decision tree and Markov model. Due to limited space, the full pathway is shown for the Advanced Life Support option only.

Figure 2. Scatterplot of the cost-effectiveness of Advanced Life Support for out-of-hospital cardiac arrest, compared to no treatment.

QALY: Quality-adjusted life years

Figure 3. Cost-effectiveness acceptability curves of different plausible treatment effects of prehospital critical care for OHCA, when compared to ALS. The underlying baseline survival to hospital discharge for the ALS cohort in this model is 9.0%.

For peer review only

For peer review only - http://bmjopen.bmj.com/site/about/guidelines.xhtml

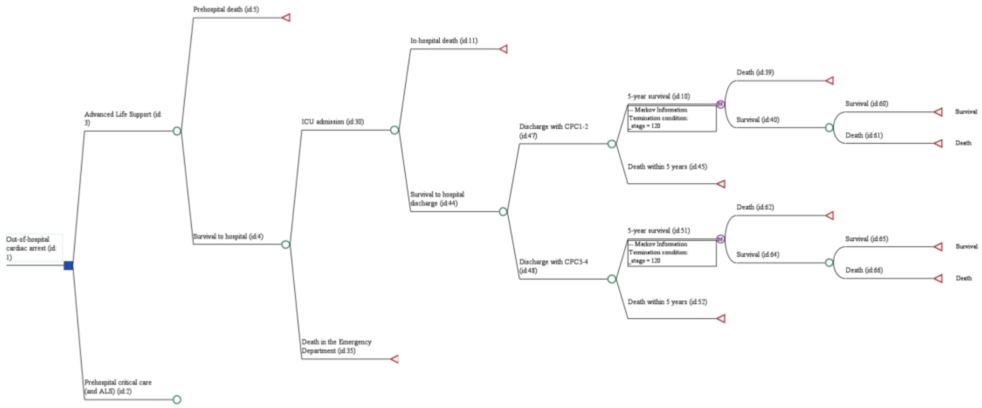

Figure 1. Final analytic model of prehospital critical care following out-of-hospital cardiac arrest, combining decision tree and Markov model. Due to limited space, the full pathway is shown for the Advanced Life Support option only.

255x110mm (300 x 300 DPI)

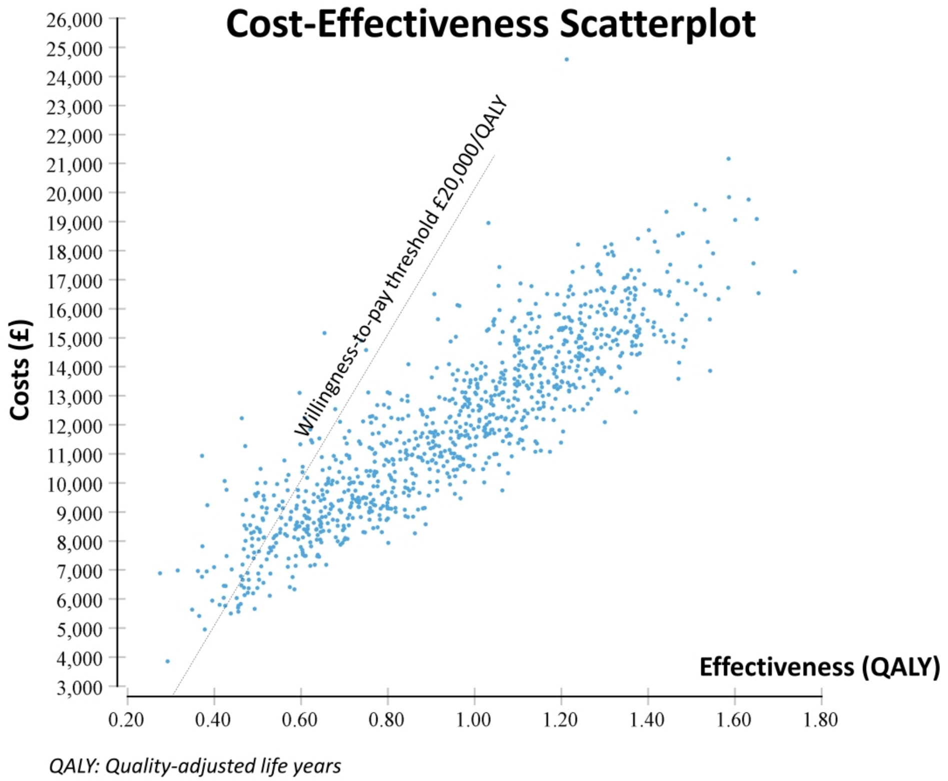

Figure 2. Scatterplot of the cost-effectiveness of Advanced Life Support for out-of-hospital cardiac arrest, compared to no treatment.

441x354mm (300 x 300 DPI)

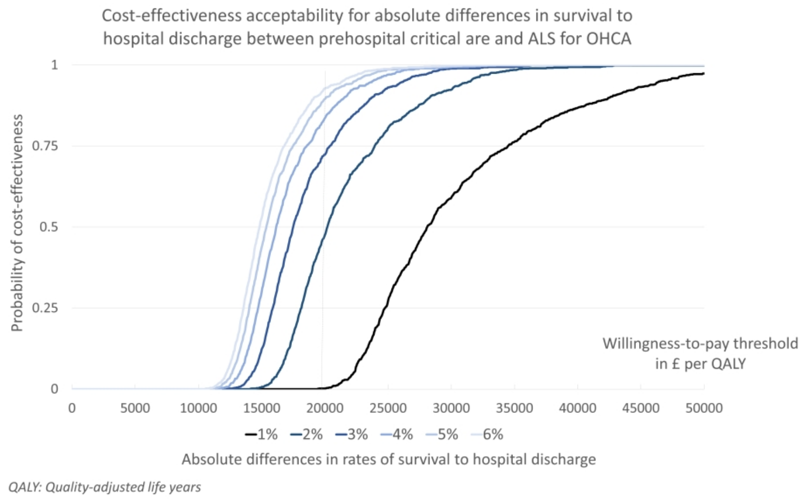

Figure 3. Cost-effectiveness acceptability curves of different plausible treatment effects of prehospital critical care for OHCA, when compared to ALS. The underlying baseline survival to hospital discharge for the ALS cohort in this model is 9.0%.

668x380mm (300 x 300 DPI)

Appendix 1. Search strategy and selection of publications for the synthesis of in-hospital costs of treatment for out-of-hospital cardiac arrest.

We undertook a focused systematic review of the literature regarding the costs of in-hospital treatment for out-of-hospital cardiac arrest (OHCA). Inclusion criteria were any descriptions of costs of in-hospital treatments in adult with non-traumatic OHCA, in the United Kingdom (UK). We did not exclude any particular method. If no data from the UK was available, data from Australia or other European countries was deemed the next best option.

PubMed was searched using the search string *(((cost) OR economics) OR expenditure) AND "cardiac arrest"*, with search results limited to the last 10 years and in English.

One reviewer undertook the screening, and selection of publications according to the above inclusion criteria. See Figure 1 for a flow chart of the search results.

Figure 1. Flow chart of the selection of publications. Search results from PubMed on 22 November 2017.

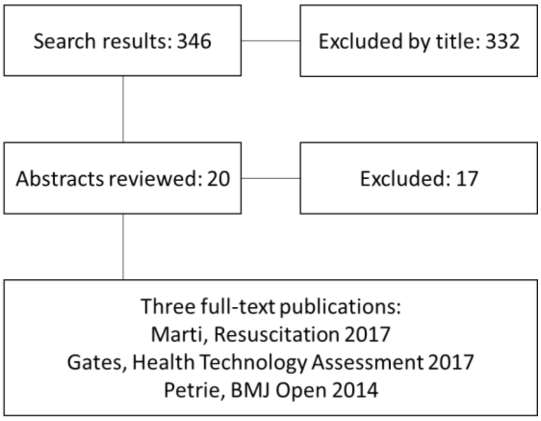

Twelve of the publications for which abstracts were reviewed were excluded as they were undertaken outside the UK, and were therefore expected to have significantly different cost and clinical parameters. The other five excluded studies did not include relevant costs. Of the three full-text publications reviewed, the studies by Marti et al. and Gates et al. related to the same randomised controlled trial of a mechanical chest compression device. Hospital treatment cost estimates were based on the length of stay of patients on the Intensive Care Unit (ICU) and on regular hospital wards as well as Emergency Department (ED) and outpatient costs. In contrast, Petrie et al. included

For peer review only

additional significant costs of cardiac interventions, such as percutaneous coronary intervention (PCI), pacemaker insertion or coronary artery bypass graft surgery (CABG), resulting in an overall higher and probably more accurate estimate of hospital costs for OHCA. We therefore used the data from Petrie et al., including the study's open access source data, in combination with the Department of Health NHS Reference Costs 2015-16, for estimating the costs of in-hospital treatment. Post-discharge costs were extracted from Gates et al.

Due to the limited available evidence and the focused nature of this review, we did not undertake a formal assessment of the risk of bias, synthesis of evidence or a detailed description of the included studies.

For peer review only

Appendix 2. Search strategy and selection of publications for the synthesis of quality and length of life after discharge from hospital following out-of-hospital cardiac arrest.

We undertook a focused systematic review of the literature regarding the length and quality of life after survival from out-of-hospital cardiac arrest (OHCA). Inclusion criteria were adult non-traumatic OHCA and outcomes of either length or quality of life measured in utility. Where available, publications using data from the United Kingdom (UK) were included; where no UK data were available, data from North America or Australia were deemed sufficient. We did not exclude any particular methods.

For peer review only

We searched PubMed, using the search string *(((quality of life[MeSH Terms]) OR utility) OR life expectancy[MeSH Terms]) AND cardiac arrest[MeSH Terms]*, with search results limited to the last 10 years.

One reviewer undertook the screening, and selection of publications according to the above inclusion criteria. See Figure 1 for a flow chart of the search results.

Figure 1. Focused literature search for data on length and quality of life following OHCA.

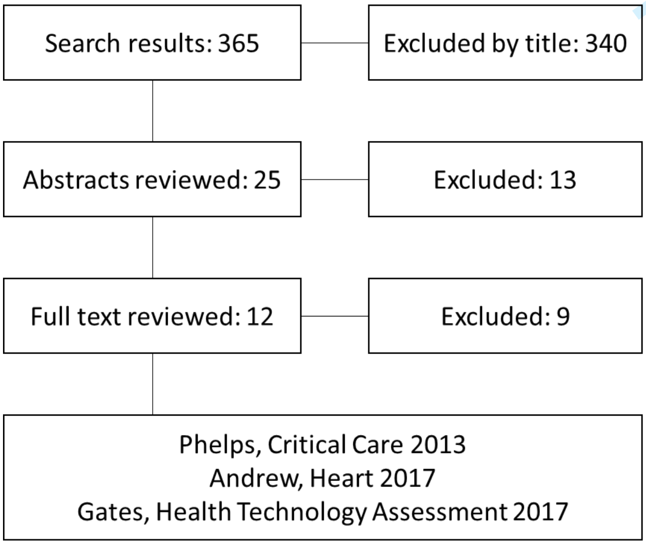

The two questions required for the decision tree and Markov model were

- What is the percentage of Cerebral Performance Category (CPC) 1-2 and CPC 3-4 amongst OHCA patients discharged from hospital?

- What is the quality and length of life of survivors of OHCA with CPC 1-2 and CPC 3-4, respectively?

Percentage of CPC 1-2 and CPC 3-4 at discharge from hospital

Gates et al. provided the most current and relevant information in their randomised controlled trial and embedded economic analysis of mechanical chest compressions during OHCA in the UK. CPC was measured at three months after discharge from hospital, with 245 of 272 (90%) of patients achieving a CPC 1-2. This is somewhat higher than the 78% and 85% of CPC 1-2 survivors reported by Petrie et al. and Phelps et al. in a UK and Australian patient population, respectively. However, both these studies measured CPC at hospital discharge, rather than at three months after discharge. Patients discharged from hospital after OHCA with CPC 3-4 have a significantly higher mortality in the first months post discharge when compared to those discharged with CPC 1-2. Additional data from Phelps et al. at twelve months post discharge demonstrate that, of the 804 patients surviving to one year post discharge, 90% were discharged from hospital with CPC 1-2. We therefore concluded that 85% was a robust estimate for the percentage of OHCA survivors discharged with CPC 1-2.

Length of life after discharge from hospital

Phelps et al. also provide the most detailed description of long-term survival following OHCA, according to CPC at discharge from hospital, including person-years for the first year and the following four years after hospital discharge. We used these data to model mortality during the first 5 years after discharge from hospital following OHCA. Andrew et al. and Phelps et al. showed that survival rates after 5 years following discharge from hospital after OHCA were the same as those of the standard population. Mortality rates were therefore obtained from the National Life Tables for England and applied to the patient cohort from five years onwards. This resulted in an overall survival rate of 65.0% and 50.0% (CPC1-2) and 36.0% and 27.8% (CPC3-4) at 10 and 15 years post discharge, respectively.

Quality of life after discharge from hospital

In their RCT which recruited a total of 4,471 patients with OHCA between 2010 and 2013 in four UK ambulance services, Gates et al. reported health-related quality of life (HRQL) using the EuroQol – 5 dimension descriptive system (EQ-5D). The EQ-5D has been validated for measuring HRQL and for its use in economic evaluations in a variety of conditions, including cardiac arrest. Gates et al. converted their findings to health-state utilities using the UK tariff. Using a combination of decision tree and Markov model similar to that employed in this research, they calculated a utility 0.75 and 0.47 for the CPC 1-2 and CPC 3-4 groups, respectively. The use of EQ-5D for the calculation of utility is recommended by the National Institute for Health and Care Excellence.

For peer review only - http://bmjopen.bmj.com/site/about/guidelines.xhtml

Due to the limited available evidence and the focused nature of this review, we did not undertake a formal assessment of the risk of bias, synthesis of evidence or a detailed description of the included studies.

For peer review only

CHEERS checklist—Items to include when reporting economic evaluations of health interventions

Section/item	Item No	Recommendation	Reported on page No/ line No
**Title and abstract**			
Title	1	Identify the study as an economic evaluation or use more specific terms such as "cost-effectiveness analysis", and describe the interventions compared.	Title, page 1
Abstract	2	Provide a structured summary of objectives, perspective, setting, methods (including study design and inputs), results (including base case and uncertainty analyses), and conclusions.	Page 2
**Introduction**			
Background and objectives	3	Provide an explicit statement of the broader context for the study.	Page 3-4
		Present the study question and its relevance for health policy or practice decisions.	Page 4
**Methods**			
Target population and subgroups	4	Describe characteristics of the base case population and subgroups analysed, including why they were chosen.	Page 5 'Clinical setting'
Setting and location	5	State relevant aspects of the system(s) in which the decision(s) need(s) to be made.	Page 5 'Clinical setting'
Study perspective	6	Describe the perspective of the study and relate this to the costs being evaluated.	Page 4 'Perspective and time horizon'
Comparators	7	Describe the interventions or strategies being compared and state why they were chosen.	Page 5 'Alternative interventions...'
Time horizon	8	State the time horizon(s) over which costs and consequences are being evaluated and say why appropriate.	Page 4 'Perspective and time horizon'
Discount rate	9	Report the choice of discount rate(s) used for costs and outcomes and say why appropriate.	Page 6 'Application of values...'
Choice of health outcomes	10	Describe what outcomes were used as the measure(s) of benefit in the evaluation and their relevance for the type of analysis performed.	Page 5-6 'Identification and synthesis...' Appendix 3
Measurement of effectiveness	11a	*Single study-based estimates:* Describe fully the design features of the single effectiveness study and why the single study was a sufficient source of clinical effectiveness data.	N/A
	11b	*Synthesis-based estimates*: Describe fully the methods used for identification of included studies and synthesis of clinical effectiveness data.	Page 5-6 'Identification and synthesis...' Appendices 2 to 3
Measurement and valuation of preference based outcomes	12	If applicable, describe the population and methods used to elicit preferences for outcomes.	Page 5-6 'Identification and synthesis...' Appendices 2 to 3
Estimating resources and costs	13a	*Single study-based economic evaluation:*Describe approaches used to estimate resource use associated with the alternative interventions. Describe primary or secondary research methods for valuing each resource item in terms of its unit cost. Describe any adjustments made to approximate to opportunity costs.	N/A

For peer review only

Section/item	Item No	Recommendation	Reported on page No/ line No
	13b	*Model-based economic evaluation:* Describe approaches and data sources used to estimate resource use associated with model health states. Describe primary or secondary research methods for valuing each resource item in terms of its unit cost. Describe any adjustments made to approximate to opportunity costs.	Page 5-6 'Identification and synthesis...' Appendices 2 to 3
Currency, price date, and conversion	14	Report the dates of the estimated resource quantities and unit costs. Describe methods for adjusting estimated unit costs to the year of reported costs if necessary. Describe methods for converting costs into a common currency base and the exchange rate.	Page 6 'Application of values...'
Choice of model	15	Describe and give reasons for the specific type of decision-analytical model used. Providing a figure to show model structure is strongly recommended.	Page 5 'Methods' Appendix 1
Assumptions	16	Describe all structural or other assumptions underpinning the decision-analytical model.	Page 5 'Methods' Appendices 1-3 Page 8 'Sensitivity analyses'
Analytical methods	17	Describe all analytical methods supporting the evaluation. This could include methods for dealing with skewed, missing, or censored data; extrapolation methods; methods for pooling data; approaches to validate or make adjustments (such as half cycle corrections) to a model; and methods for handling population heterogeneity and uncertainty.	Page 7, Table 1 Page 8 'Sensitivity analyses'
**Results**			
Study parameters	18	Report the values, ranges, references, and, if used, probability distributions for all parameters. Report reasons or sources for distributions used to represent uncertainty where appropriate. Providing a table to show the input values is strongly recommended.	Table 1
Incremental costs and outcomes	19	For each intervention, report mean values for the main categories of estimated costs and outcomes of interest, as well as mean differences between the comparator groups. If applicable, report incremental cost-effectiveness ratios.	Figure 2, Table , Table 4. We chose median rather than mean, due to the considerably skewed distributions
Characterising uncertainty	20a	*Single study-based economic evaluation* :Describe the effects of sampling uncertainty for the estimated incremental cost and incremental effectiveness parameters, together with the impact of methodological assumptions (such as discount rate, study perspective).	N/A
	20b	*Model-based economic evaluation:* Describe the effects on the results of uncertainty for all input parameters, and uncertainty related to the structure of the model and assumptions.	Page 7, Table 1
Characterising heterogeneity	21	If applicable, report differences in costs, outcomes, or cost-effectiveness that can be explained by variations between subgroups of patients with different baseline characteristics or other observed variability in effects that are not reducible by more information.	Figure 2, Table 3, Table 4
Study findings, limitations, generalisability, and	22	Summarise key study findings and describe how they support the conclusions reached. Discuss limitations	Page 12-14

For peer review only

Section/item	Item No	Recommendation	Reported on page No/ line No
current knowledge		and the generalisability of the findings and how the findings fit with current knowledge.	
**Other**			
Source of funding	23	Describe how the study was funded and the role of the funder in the identification, design, conduct, and reporting of the analysis. Describe other non-monetary sources of support.	Page 3
Conflicts of interest	24	Describe any potential for conflict of interest of study contributors in accordance with journal policy. In the absence of a journal policy, we recommend authors comply with International Committee of Medical Journal Editors recommendations.	Page 3

For consistency, the CHEERS statement checklist format is based on the format of the CONSORT statement checklist

For peer review only
